# Supplementary material for: Fungus-originated glucanase and monooxygenase genes in creeping bent grass (Agrostis stolonifera L.)
Source: PLoS One. 2021 Sep 10;16(9):e0257173. doi: 10.1371/journal.pone.0257173 (PMC8432771; doi:10.1371/journal.pone.0257173)
Supplement: S1 Fig — mRNA sequences from E. amarillans and creeping bent grass are aligned with a part of genomic sequence from perennial ryegrass. A dash (-) shows a gap in the DNA sequences, and an asterisk (*) under the alignment denotes ‘conserved nucleotide’. The intron sequence of perennial ryegrass glucanase-like gene is shown with an empty box. The location and direction of each PCR primer are shown with a blue arrow. (PDF) [file pone.0257173.s001.pdf]

S1 Fig. DNA sequence alignment of the glucanase(-like) genes.

```
E. amarillans            ------------------------------------------------------------
Creeping bent grass      GCACTTACTTGTCCATGAAAGTCACGACGATAAGGCCGTCTGACGGGGCACAGATTCTAT
Perennial ryegrass       GCACTTACTTGTCCATCAAAGTCATGACGATAAGCCCGTCTGACGGGGCACAGATTCTAT

E. amarillans            --------------------------ATGCATCATTCTATCCTTGTCCC-GGCGCTCCTG
Creeping bent grass      AAAAAGGCCAGCTGTGAACATCACCCACACCGCGCCTAATCCTCTCCTCTAGAGCGCTCA
Perennial ryegrass       AAAAAGGCCAGCTGTGAACATCACCCTCACCGCGCCTAATCCTCTCCTCTAGAGCTCTCA
                                                      *  *     *****   * *  * ** *

E. amarillans            GCCGGTGCCGCCTCGGCCTGGCTTCCC-----CAGGAGCGCGACTTGGCTGCTTTCAACC
Creeping bent grass      GCAGCAGCAGACGAGATGCATCATTCTATCATTATTATCCCGACGTTGGCGCTCCTGGCC
Perennial ryegrass       CC---AATAGACGAGATGCATCGTTCTATCGTTGGTATCACGACGCTGGCGCTCCTGGCC
                          *       * *  *      * * *          * * ****   *  ***     **

E. amarillans            AGACGGCTCGCTTCGAACAGCTTGGCAAGCGCTTCGCGCCGTCTCTGCCCAAGGGCATCA
Creeping bent grass      GG--TGCTGTCTCGGCCTGGCTTCCCAAGGAGCACGACTTGGCTGCTTTCAAGGGCATCA
Perennial ryegrass       GG--TACTGCCTCGGCATGGCTTCCCAAGGAGCACGACTTGGCTGCTTTCAAGGGCATCA
                          *    **  **  *    ****  ****     **    * **     ***********

E. amarillans            ACAAGATCAGGGGCGTTAATTTCGGCGG--------------------------------
Creeping bent grass      ACAAGATCAGGGGCGTTAATTTCGGCGG--------------------------------
Perennial ryegrass       ACAAGATCAGGGGCGTTAATTTCGGCGGTATGTCTTCTTTACAGCTGCTACTTCCTCCAT
                         ****************************
                         AsBGNL_exon_F

E. amarillans            ------------------------------------------------------------
Creeping bent grass      ------------------------------------------------------------
Perennial ryegrass       CCTCCGATAATACAAGTCACATTCTTTTTGCAAAATTATATGGATCCAGAAGTATAAATG
```

S1 Fig. (Cont'd)

```
E. amarillans           ------------------------------------------------------------
Creeping bent grass     ------------------------------------------------------------
Perennial ryegrass      GTTGCATTCCTCTTACAGATCTCTTTCATTTCATATATGTTCATTCAGATTTTGTGGAGT

E. amarillans           -----------------------------CTGGCTCATCTGTGAGCCGTGGATGATGAGT
Creeping bent grass     -----------------------------CTGGCTCGTCTGTGAGCCGTGGATGATGAGC
Perennial ryegrass      ACTAACTAACAGAGGTTGAAATTCTCAGGCTGGCTCATCTGTGAGCCATGGATGATGAGC
                                                     ******* ********** *********** 
                                                   AsBGNL_intron_F    AsBGNL_exon_R

E. amarillans           GACGAGTGGAACAACGTCATGGGTTGCAACGGGGCTGCCTCCGAGTTCGACTGCATGCGA
Creeping bent grass     GACGAGTGGAACAACGTCATGGGCTGCAACGGGGCTGCCTCCGAGTTCGACTGCATGCTA
Perennial ryegrass      GACGAGTGGAACAACGTCATGGGCTGCAACGGGGCTGCCTCCGAGTTCGACTGCATGCTA
                        *********************** ********************************** *
                                                              LpBGNL_cons_F

E. amarillans           AACAATTACGGTGGAAGTAAACGAGACGCAGGCAACGAGAAGTTCGAGACTCACTGGAGG
Creeping bent grass     AACAATTACATGGGAAGCAAACGAGACGCAGGCAACGAAAAGTTCGAGGCTCATTGGAGG
Perennial ryegrass      AACAATTACATGGGAAGCAATCGAGCTGCCGGCAACGACAAGTTCGAGACTCATTGGAGG
                        *********   ***** ** ****  ** ******** ********* **** ******

E. amarillans           ACCTGGATCAATGCCGACAGCGTCCAGTCAGCCCACGACGTTGGCCTGAACACGCTTCGC
Creeping bent grass     ACTTGGATCAATGCCGACAGCGTCGAGTCAGCCCACTACGTTGGCCTGAACACGATTCGC
Perennial ryegrass      ACTTGGATCAATGCCGACAGCGTCGAGTCAGTCCACTATGTTGGCCTGAACACGATTCGC
                        ** ********************* ****** **** * *************** *****

E. amarillans           ATTCCCATGGGGTACTGGTCCTACGTAGACATTGTCGACAAGGGCAGCGAACCCTTTGCC
Creeping bent grass     ATTCCCATCGGGTACTGGTCCTACGTAGACATTGTCGACAAGGCCAGCGAGCCCTTTGCC
Perennial ryegrass      ATCCCCATCGGGTACTGGTCCTACGTGGACATTGTCGACAAGGCCAGTGAGCCCTTTGCC
                        ** ***** ***************** **************** *** ** *********
```

S1 Fig. (Cont'd)

```
E. amarillans          GACGGCAACAAGATGCTCCCCTACCTGGACGCCGTCGTCCAAAAGGCCGCTGACCTCGGC
Creeping bent grass    GACGGCAACAGGATGCTCCCCTACCTGGACGCCGTCGTCCAAAAGGCTGCTGATCTTGGC
Perennial ryegrass     GATGGCAACAGGATGCTCCCATACCTGGACGCCGTCGTCCAAAAGGCTGCTGACCTTGGC
                       ** ******* ********* ************************** ***** ** ***


E. amarillans          ATGTATGTCATCATCGATCTGCACGGGGCCCCCGGCGGCCAGCAAGAAGACGTCTTTACC
Creeping bent grass    ATGTATGTCATCATCGATCTGCACGGGGCCCCCGGTGGCCAGCAACAAGACGTCTTTACC
Perennial ryegrass     ATGTATGTCATCATCGATCTTCATGGGGCTCCCGGCGGGCAGCAACAAGACGCCTTCACC
                       ******************** ** ***** ***** ** ****** ****** *** ***


E. amarillans          GGCCAGAACAACAAGCCGGCCGGTTTCTTCAACGACTACAACTTTGACCGTGCCCAGAAG
Creeping bent grass    GGCCAAAACAACAAGCCGGCCGGTTTCTTCAACGACTACAACTTTGACCGCGCCCAGAAG
Perennial ryegrass     GGCCAGAACAACAAGCCGGCCGGTTTCTTCAACGACTACAACTTTGACCGCGCCAACAGG
                       ***** ******************************************** *** * * *


E. amarillans          TGGATGTCGTGGATGACGAAGCGCATCCACACAAACCCTGCCTACGCCACCGTCGGCATG
Creeping bent grass    TGGATGTCGTGGATGACGAAGCGCATCCACACAAACCCTGCCTACGCAACCGTCGGCATG
Perennial ryegrass     TGGATGTCGTGGATGACGAGGCGCATCCACACAAACCCTGCCTATGCCACCGTCGGCATG
                       ******************* ************************ ** ************
                                       LpBGNL_cons_R

E. amarillans          ATTGAGGTTCTCAACGAGCCCGTCTCCGGGCACGACGGGGGCGGACGGTACCCTGCCCCC
Creeping bent grass    ATTGAGGTTCTCAACGAGCCCGTCTCCGGGCACGACCCGGACCGGCGGTACCCTGCCCCT
Perennial ryegrass     ATTGAGGTTCTCAACGAGCCCGTGTCCGGACACGACTCGGACGGACGGTACCCTGCCCCC
                       *********************** ***** ******  ** * * ************** 
                       AsBGNL/LpBGNL_F

E. amarillans          GGTGAGGTCCCCGGGCTGGTCCAGAAGTACTACCCGGGCGCTCTGAAGGCGGTCCGAGAT
Creeping bent grass    GGTCAGGTCCCTGGGCTAATCCACAAGTACTACCCGGGCGCTCTCAAGGCGGTCCGAGAT
Perennial ryegrass     GGTCAGGTCCCCGGGCTGGTCCAGAAGTACTACCCGGGCGCTCTCAAGGCGGTCCGAGAT
                       *** ******* *****  **** ******************** ***************
```

S1 Fig. (Cont'd)

```
E. amarillans          GCCGAAGCGTCGCTCGGCGTGGCTGACGGCAAGAAGCTCCACGTGCAATTCATGTCCCAG
Creeping bent grass    GCCGAAGCGTCGCTCGGCGTGGCTGACGGCAGCAAGCTCCACGTGCAATTCATGTCCCAG
Perennial ryegrass     GTCGAAGCGTCTCTCGGAGTGGCTGACGGCAACAAGCTCCATGTGCAGTTCATGTCCCAG
                       * ********* ***** *************  ******** ***** ************
                                                                          AsBGNL_R
                                                                          LpBGNL_R

E. amarillans          AAATGGGACTCGGGCAACCCGCGCGACAACTCTGCCGTGGCCAACGACAAGCTGACTGCG
Creeping bent grass    AAATGGGACTCGGGCAACGCGCGCGACAACTCTGCCGTGGCCAACGACAAGCACACTGCG
Perennial ryegrass     AAGTGGGACTCGGGCAACGCGCGCGACAACTCTGCCGTGGCCAACGACCAACGCACTGCG
                       ** *************** ***************************** * *  ******

E. amarillans          TTCGATGACCACAACTACATTGGCTTTGCCGTCAAGGACAACGGCAACCGGGACAAGCTC
Creeping bent grass    TTCGATGACCACAACTACATTGGCTTCGCCGTCGGCGACAGGGGCAACCGGGACAGCCTC
Perennial ryegrass     TTCGATGACCACAACTACATTGGCTTCGCATTGAAAGACGGGGGCGACCGGGACAGCCTC
                       ************************** **  *    ***   *** *********  ***

E. amarillans          ATGAAGTCGGCCTGCAGGGACAATCGCGTCGTGGACGGGCAGACGTTCGCCATTACCGGC
Creeping bent grass    ATGAGGAGTGCCTGCAATGACCATCGCACCGTGAACGGGCAGACGTTCGCCATTACCGGC
Perennial ryegrass     ATGAGGAGCGCCTGCAATGACCATCGCACCGTGAACGGGCAGGCATTCACCATTACCGGC
                       **** *   *******  *** *****  **** ******** * *** ***********

E. amarillans          GAATGGAGCATGACATCGGACGTGAGCCCCGACGACACAGACTTCTTCAAGAAGTTCTTC
Creeping bent grass    GAGTGGAGCATGACATCGGACGTGAGCCCGGACGACGCAGACTTCTTCACCAAGTTCTTC
Perennial ryegrass     GAGTGGAGCATGACATCGAGCGTGAGCCCGGACGACAAAGACTTCTTCAGGAAGTTCTTC
                       ** ***************  ********* ******  ***********  *********

E. amarillans          ACGGCCCAACAGCAGCTCTACGAGGCGCCTGGGATGAGTGGCTGGGTGTACTGGACGTGG
Creeping bent grass    ACGGCCCAACAGCAGCTCTACGAGGAGCCTGGGATGAGTGGCTGGGTCTACTGGACGTGG
Perennial ryegrass     ACGGCCCAACAACAGCTCTACGAGGAGCCTGGGATGAGTGGCTGGATCTACTGGACGTGG
                       *********** ************* ******************* * ************
```

S1 Fig. (Cont'd)

```
E. amarillans          AAGACGCAGTTGAATGATCCTCGCTGGACCTACTCTGACGCCACGTACCGCAAACTGATC
Creeping bent grass    AAGACGCAGTTGAATGATCCTCGGTGGACCTACTCTCACGCCACGTACCTCAAACTGGTC
Perennial ryegrass     AAGACGCAGTTGAATGATCCTCGATGGACCTACTCTCACGCCACGTACCTCAATCTGATC
                       *********************** ************ ************ *** *** **

E. amarillans          CCCACCGATGCCGTTGGCTTGGAAAGGAATGTGTATCAAGATGTCTGTTCCAGTTATAGA
Creeping bent grass    CCCACCGATGCCGCTGCCTTGGAAAGGAATGTGTATCAAGATGTGTGTTACCCTTATAGA
Perennial ryegrass     CCCACAGATGCCGCTGCCCTGGAAAGGAATGTGTATCAAGATATCTGTTACCCTTATAGA
                       ***** ******* ** * *********************** * **** *  *******

E. amarillans          TAG---------------------------------------------------------
Creeping bent grass    TAGAGAGACACGTACTATTTTATTTTGAGATATTTACCCACCACTGCCACCGGTAAGTCA
Perennial ryegrass     TAGAGACACATACTAAGTACGACTTTTTAGATGAGGTCTATTAATACCTCTTATATTTTA
                       ***

E. amarillans          ------------------------------------------------------------
Creeping bent grass    ATAACGGTGAGTTTGACGACGATGAGGTCGTCAAGATCAGACCGCCCACGTCAGGGTTGT
Perennial ryegrass     CTAATGTGCCGTCAGTTTTCCCCTTAAGCGTG-----CAAATTTCATAATTTGGGATTTT

E. amarillans          ------------------------------------------------------------
Creeping bent grass    TGTTGCTGCCGCATCGTTCCAGGTTCCTATGGTCAGATGAGTGTGAAGCTAGCTTCAACA
Perennial ryegrass     AATTTCCATGAGGAAATTGGAAATTGT---------------------------------

E. amarillans          -------------------------------------------
Creeping bent grass    AGCCAAAAACCCGTTTGACTACCGCACCCGTCTTGGTACTTCC
Perennial ryegrass     -------------------------------------------
```
