# Supplementary material for: Fungus-originated glucanase and monooxygenase genes in creeping bent grass (Agrostis stolonifera L.)
Source: PLoS One. 2021 Sep 10;16(9):e0257173. doi: 10.1371/journal.pone.0257173 (PMC8432771; doi:10.1371/journal.pone.0257173)
Supplement: S2 Fig — The AsFMOL sequence is aligned with E. amarillans and E. festucae FMO sequences. A dash (-) shows a gap in the DNA sequences, and an asterisk (*) under the alignment denotes ‘conserved nucleotide’. The location and direction of each PCR primer are shown with a blue arrow. The AatII restriction enzyme recognition site in the E. amarillans and E. festucae sequences is indicated with an empty box. (PDF) [file pone.0257173.s002.pdf]

# S2 Fig. DNA sequence alignment of the flavin-containing monooxygenase(-like) genes

```
E. amarillans            ------------------------------------------------------------
E. festucae              ------------------------------------------------------------
Creeping bent grass      CTAATAGTCACGCCTACCTCAGTGTTATTCATTTGTCGAGGAACCCGCGAAGCAAGCATG


E. amarillans            ------ATGGCTCCCCGACGCCAGGATGCGAGTGTTGCGGACTATGATGTCGTCGTCGTC
E. festucae              ------ATGGCTCCCCGACGCCAGGATGCGATTGTTGCGGACTATGATGTCGTCATCGTC
Creeping bent grass      CATAGCATGGCTCCCCGACGCCAGGATGCCATTGTTGCGGACTATGATGTCGTCATCGTC
                               *********************** * ********************** *****

E. amarillans            GGCGCGGGCATCTCCGGCATCAACTTCGCGTACAGGCTGCAGGAGCGCAGCCCGGAGCTG
E. festucae              GGCGCGGGCATCTCCGGCATCAATTTCGCGTACAGGCTGCAGGAGCGCAACCCGGAGCTG
Creeping bent grass      GGCGCGGGCATCTCCGGCATCAATTTCGCGTACCGGCTGCAGGAGCGCAACCCGGAGCTG
                         *********************** ********* *************** **********

E. amarillans            AGCTACTGCATCCTCGACGGGCGGCACCAGGTCGGCGGGACGTGGAGCCTGTTCCAGTAC
E. festucae              AGCTACTGCATCCTCGAGGGGCGGCACCAGGTCGGCGGGACGTGGAGCCTGTTCCGGTAC
Creeping bent grass      AGCTATTGCATCCTCGAGGGGCGGCACCAGGTCGGCGGGACGTGGAGCCTGTTCCAGTAC
                         ***** *********** ************************************* ****

E. amarillans            CCGGGCATCCGGTCCGATTCCGACCTGTACACGTTCGGCTTCCCCTGGCGGCCGTGGGAG
E. festucae              CCGGGCATCCGGTCCGATTCCGACCTGTACACGTTCGGCTTCCCCTGGCGGCCGTGGGAG
Creeping bent grass      CCGGGCATCCGGTCCGATTCCGACATGTACACGTTCGGCTTCCCCTGGCGGCCGTGGGAG
                         ************************ ***********************************

E. amarillans            GAGAAGCAGCCCATCGCCCGCGGCTCGCTCATCCTCAAGTACCTCGAGGCGTCTGCTGCC
E. festucae              GAGAAGCAGCCCATCGCCCGCGGCTCGCTCATCCTCAAGTACCTCGAGGCGTCTGCCACC
Creeping bent grass      GAGAAGCAGCCCATCGCCCGCGGCTCGCTCATCCTCAGGTACCTCGAGGCGTCTGCCGCC
                         ************************************* ******************  **
```

## S2 Fig. (Cont'd)

```
E. amarillans           GAGGCGGGCATCGACAACAAGATCAGGTTCAACCATCGGGTCGACAGCATGGACTGGTCC
E. festucae             GAGGCGGGCATCGACAACAAGATCAAGTTCAACCACCGGGTCGACAGCATGGACTGGTCC
Creeping bent grass     GAGGCGGGCATCGACAACAAGATCAAGTTCAACCATCGGGTCGACAGCATGGACTGGTCC
                        ************************* ********* ************************


E. amarillans           TCGGCGTCGTCGACCTGGACGCTCAACGTGGCGGCCGGCGGCGGCGGCGACAACGTCATC
E. festucae             TCGGCGTCGTCGACCTGGACGCTCAACGTGACGGCTGGCGGCGGCGGCG------TCGTC
Creeping bent grass     TCGGCGTCGTCGACCTGGACGCTCAACGTGACGGCCGACAACGTCGTCT------CCCTG
                        ****************************** **** * *  ** ** *        * *


E. amarillans           TCCCTGCGCGCCCGGTTCGTGCTGCTCGGCACGGGATACTACGACTACGACGAGCCGCTC
E. festucae             TCCATGCGCGCCCGGTTCGTGCTGCTCGGCACGGGATACTACGACTACGACGAGCCGCTC
Creeping bent grass     CGCCTGCGCGCCCGGTTCGTGCTGCTCGGCACGGGATACTACGACTACGACGAGCCGCTC
                          * ********************************************************


E. amarillans           GAGGCCCGCATCCCCGGCATAGACAGGTTCCGGGGCGCCGTCGTCCACCCCCAGTTCTGG
E. festucae             GAGGTCCGTATCCCCGGCATAGAAAGGTTCCGGGGCGCCGTCGTCCACCCCCAGTTCTGG
Creeping bent grass     GAGGCCCGTATCCCCGGCATAGACAGGTTCCGGGGCGCCGTCGTCCACCCCCAGTTCTGG
                        **** *** ************** ************************************


E. amarillans           CCCCGGGACCTGGACTACACCGGCAAGAACGTCGTCGTCATCGGGTCCGGCGCCACCGCC
E. festucae             CCCCGGGACCTGGACTACACCGGCAAGAACGTCGTCGTCATCGGGTCCGGCGCCACCGCC
Creeping bent grass     CCCCGGGACCTGGACTACACCGGCAAGAACGTCGTCGTCATCGGGTCCGGCGCCACCGCC
                        ************************************************************


E. amarillans           GTCACGCTGCTCCCCAGCATGGCCGACAAGGCCTCGCACACGACCATGCTGCAGCGGTCG
E. festucae             GTCACGCTGCTCCCCAGCATGGCCGACAAGGCCGCACACACGACCATGCTGCAGCGGTCG
Creeping bent grass     GTCACGCTGCTCCCCAGCATGGCCGACAAGGCCGCGCACACGACCATGCTGCAGCGGTCG
                        ********************************* * ************************
```

## S2 Fig. (Cont'd)

```
E. amarillans          CCCAC------------------------------------------------GGCCGCC
E. festucae            CCCACGTACATCCTCGCCCTGCCCCAAAACGACC---------CTCTCGACAGGGCCGCC
Creeping bent grass    CCCACGTACATCCTCGCCCTGCCCGAAAACGACGACCCTGCTGCTCTCGACAGGGCCGCC
                       *****                                                *******

E. amarillans          CGGCTCCTGCTCCCCGGCGCGCTGGCCAGGCGACTGATTCGCTTCAGGTGGATCCTGTCG
E. festucae            CGGCTCTTGCTCCCCGGCGCGCTGGCCAGGCGACTGATTCGCTTCAGGTGGATCCTGTCG
Creeping bent grass    CGGCTCTTGCTCCCCGGCGCGCTGGCCGGGCGACTGATTCGCTTCAGGTGGATCCTGTCG
                       ****** ******************** ********************************

E. amarillans          GCGTTTCTTCTCGCAAACCTGTGCCGGTGGTTCCCGCGGCTCGCCCGCGCCGTCATCCTC
E. festucae            GCGTTTCTTCTCGCAAACCTGTGCCGGTGGTTCCCGCGGCTCGCCCGCGCCGTCATCCTC
Creeping bent grass    GCGTTTCTTCTCGTAACCCTGTGCCGGTGGTTCCCGCGGCTCGCCCGCGCCGTCATCCTC
                       ************* ** *******************************************
                         AsFMOL_SCA_f1

E. amarillans          CGGCGGACGTCCCGCGAGCTGCCGAGCGGCACCGGGATGAAGCCTCACTTCACCCCGCGG
E. festucae            CGGCGGACGTCCCGCGAGCTGCCGAGCGGCACCGGGATGAAGCCTCACTTCACCCCGCGG
Creeping bent grass    CGGCGGACGTCCCACGAGCTGCCGAGCGGCACCGGGATGAAGCCTCACTTCACCCCGCGG
                       ************* **********************************************

E. amarillans          TACAACCCCTGGGAGCAGCGCATGTGTATCTGCCCGGGCGGCGACTTCTTCCAGTGCCTG
E. festucae            TACAACCCCTGGGAGCAGCGCATGTGTATCTGCCCGGGCGGCGACTTCTTCCAGTGCCTG
Creeping bent grass    TACAACCCCTGGGAGCAGCGCATGTGTTTCTGCCCGGGCGGCGACTTCTTCCAGTGCCTG
                       *************************** ********************************

E. amarillans          CGCGCCGGCAAGGCCAGCGTGGAGACGGGCGTCATCGAGCAAGTCACCGAGGACACCATC
E. festucae            CGCGCCGGCAAGGCCAGCGTGGAGACGGGCGTCATCGAGCAAGTCACCGACAACACCATC
Creeping bent grass    CGCGCCGGCAAGGCCAGCGTGGAGACGGGCGTCATCGAGCAAGTCACCGAGAACACCATC
                       **************************************************  ********
                                                    AsFMOL_con_f1_AstII
```

## S2 Fig. (Cont’d)

```
E. amarillans         AGGCTCGAGTCCGGCCGGGAGCTGCACCCAGACATCATCGTCACCGCCACGGGTTTGAAG
E. festucae           AGGCTCGAGTCCGGCCGGGAACTGCACCCAGACATCATCGTCACCGCCACGGGGTTGAAG
Creeping bent grass   AGGCTCGAGTCCGGCCGGGAACTGCACCCGGACATCATCGTCACCGCCACGGGGTTGAAG
                      ******************** ******** *********************** ******

E. amarillans         GTCCGGCTCGCGGGGGGCATCGACGTCTCTCTCGACGGCAGGCCCGTCCGCGTGCCCGAC
E. festucae           GTCCGGCTCGCGGGGGGCATCGACGTCTCTTTCGACGGCAGGCCCGTCCGCGTGCCTGAC
Creeping bent grass   TTCCGGCTCGCGGGGGGCATCGGCATCTCTCTCGACGGCAGGCCCGTCCGCGTGCCCGAC
                       ********************* * ***** ************************* ***

E. amarillans         TGCTACTCGTGGAAGGGGTGCATGCTCGAGAACCTGCCCAACCTGTTCTATTCCATGGGC
E. festucae           TGCTACTCGTGGAAGGGGTGCATGCTCGAGAAACTGCCCAACTTGTTCTATTCCATGGGC
Creeping bent grass   TGCTACTCGTGGAAGGGGTGCATGCTCGAGAACCTGCCCAATCTGTTCTATTCCATGGGC
                      ******************************** ********  *****************
```

AsFMOL_con_r1_AstII

```
E. amarillans         TACGTCGACGCGAGCTGGACGCTCGGCGCCGACGCCACCGCCCAGCTCGTCTGCCGCCTT
E. festucae           TACGTCGACGCGAGCTGGACGCTCGGCGCCGACGCCACCGCCCAGCTCGTCTGCCGCCTT
Creeping bent grass   TACGTCGACGCGAGCTGGACGCTCGGCGCCGACGCCACCGCCCAGCTCGTCTGCCGCCTT
                      ************************************************************

E. amarillans         CTGAACAGGATCACGCGCGACGGCTTCGCGCACATAGTGCCCCGGCTGAGCGAGGCGGAA
E. festucae           CTGAACAGGGTCACGCGCGACGGCTTCGCGCACATAGTGCCCCGGCTGAGCGAGGCGGAA
Creeping bent grass   CTCAACAGGGTCACGCGC---------------ATAGTGCCCCGGCTGAGCGAGGCGGAA
                      ** ****** ********               ***************************
```

AsFMOL_ps_f1

```
E. amarillans         AAGAGCACCATGTCCGAGCTGCCCTTTCTGCGTCTCAACTCAACCTACGTGGACAGGGGC
E. festucae           AAGAGCACCATGTCCGAGCTGCCTTTTCTGCGTCTCAACTCAACCTACGTGGACAGGGGC
Creeping bent grass   AAGAGCACCATGTCCGAGCTGCCCTTTCTGCGTCTCAACCCAACCTACGTGGACAGGGGC
                      *********************** *************** ********************
```

# S2 Fig. (Cont'd)

```
E. amarillans          AAGGGCGTCTTTCCCAAGGTGGGAAACAGCAAGCAGTGGCTTCCGCGATCATACTACTGG
E. festucae            AGGGGCGTCTTTCCCAAGGTGGGAAACAGCAAGCAGTGGCTTCCGCGATCATACTACTGG
Creeping bent grass    AAGGGCGTCTTTCCCAAGGTGGGAAACAGCAAGCAGTGGCTTCCACGATCATACTACTGG
                       * ****************************************** ***************
                                                      AsFMOL_ps_r1 <---------------
                                                            AsFMOL_SCA_r1 <-

E. amarillans          AAGGATCTCGCGAATGCGTGGTGGGGAGACGTGCAAAGCGGATTGGAATGGTCAAGATAG
E. festucae            AAGGATCTCGCGAATGCGTGGTGGGGAGACGTGCAAAGCGGGTTGGAATGGTCAAGATAG
Creeping bent grass    AAGGATCTCACGAATGCGTGGTGGGGAGACGTGCAAAGCGGCTTGGAATGGTCAAGATAG
                       ********* ******************************* ******************

E. amarillans          ---------------
E. festucae            ---------------
Creeping bent grass    CTTTTTTTTTCATCA
```
