# Supplementary figures and images for: Fungus-originated glucanase and monooxygenase genes in creeping bent grass (Agrostis stolonifera L.)
Source: PLoS One. 2021 Sep 10;16(9):e0257173. doi: 10.1371/journal.pone.0257173 (PMC8432771; doi:10.1371/journal.pone.0257173)

**S3 Fig. PCR-based screening using the *AsBGNL* and *LpBGNL* locus-specific primers**

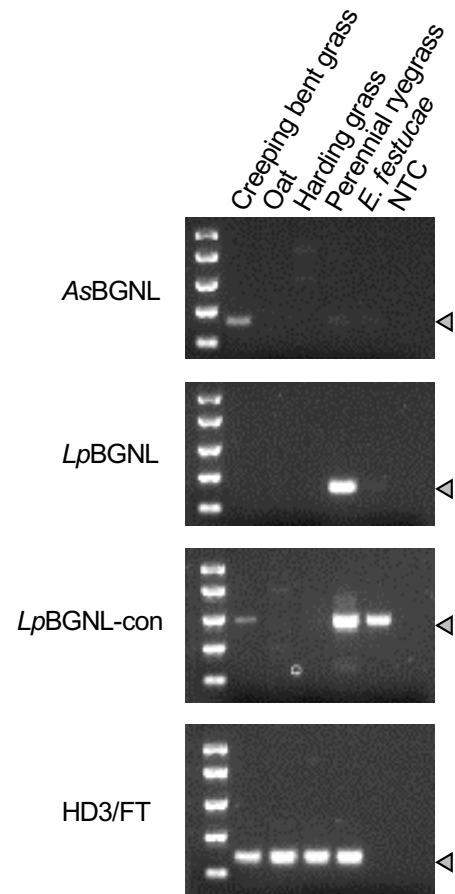

Supplement: S3 Fig — The gDNA samples of creeping bent grass, oat, harding grass, perennial ryegrass, and E. festucae were used as DNA template. A control experiment with the florigen candidate gene (HD3/FT)-specific primers was performed, to confirm the quality of plant gDNA samples. NTC stands for ‘no template control’. The PCR amplicons were visualised on an agarose gel (2% w/v) containing the SYBR Safe stain, and the expected size of PCR amplicons is indicated with a grey-filed arrow. The BIOLINE EasyLadder I was used as size standard. (PDF) [file pone.0257173.s003.pdf]

**S4 Fig. qPCR amplification plot for the AsFMOL gene expression analysis**

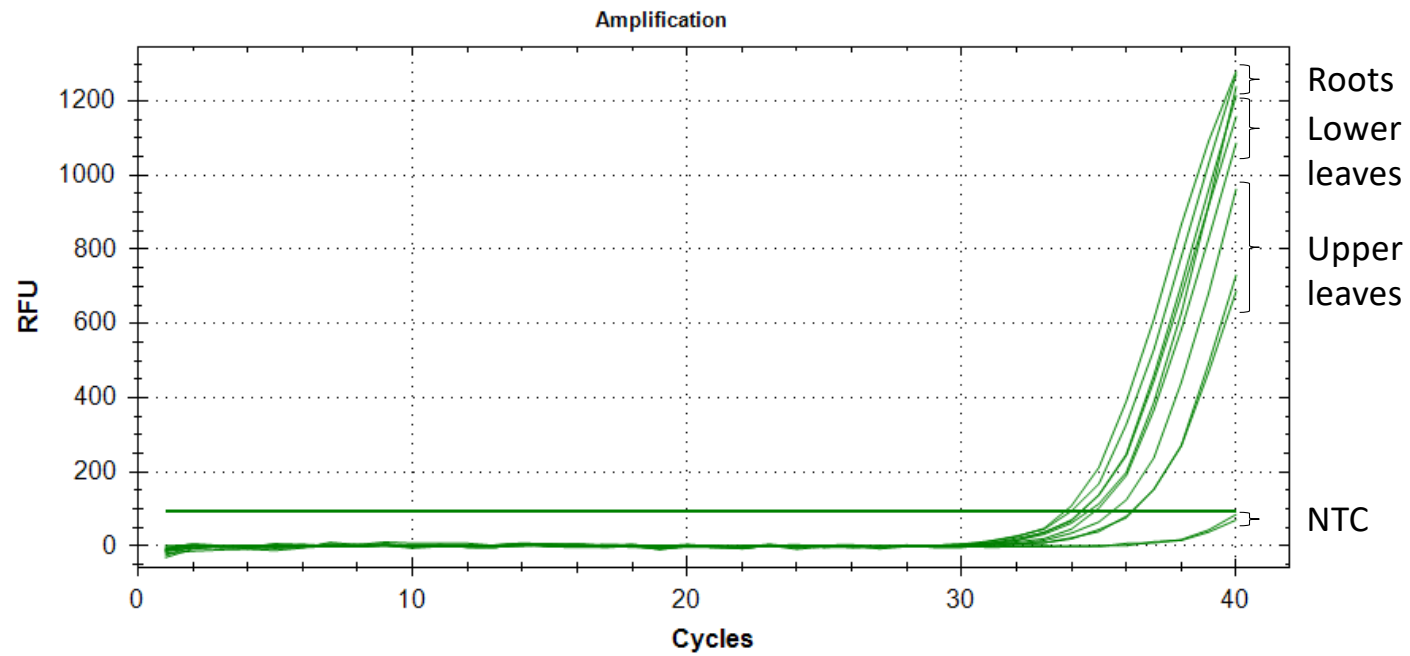

Supplement: S4 Fig — The vertical and horizontal axes indicate the relative fluorescence units (RFU) and the number of PCR cycles. cDNA samples from roots, lower leaves, and upper leaves were used as DNA template. NTC stands for ‘no template control’. The sample names are shown on the right side of the plot. The threshold line is shown with the thick green line. The plot was generated with CFX Maestro Software (BioRad). (PDF) [file pone.0257173.s004.pdf]
