# Supplementary material for: Fungus-originated glucanase and monooxygenase genes in creeping bent grass (Agrostis stolonifera L.)
Source: PLoS One. 2021 Sep 10;16(9):e0257173. doi: 10.1371/journal.pone.0257173 (PMC8432771; doi:10.1371/journal.pone.0257173)

S5 Fig. PCR assay with the primers designed across the intron (upper panel) and on the exon/intron boundaries (lower panel) of *As*BGNL

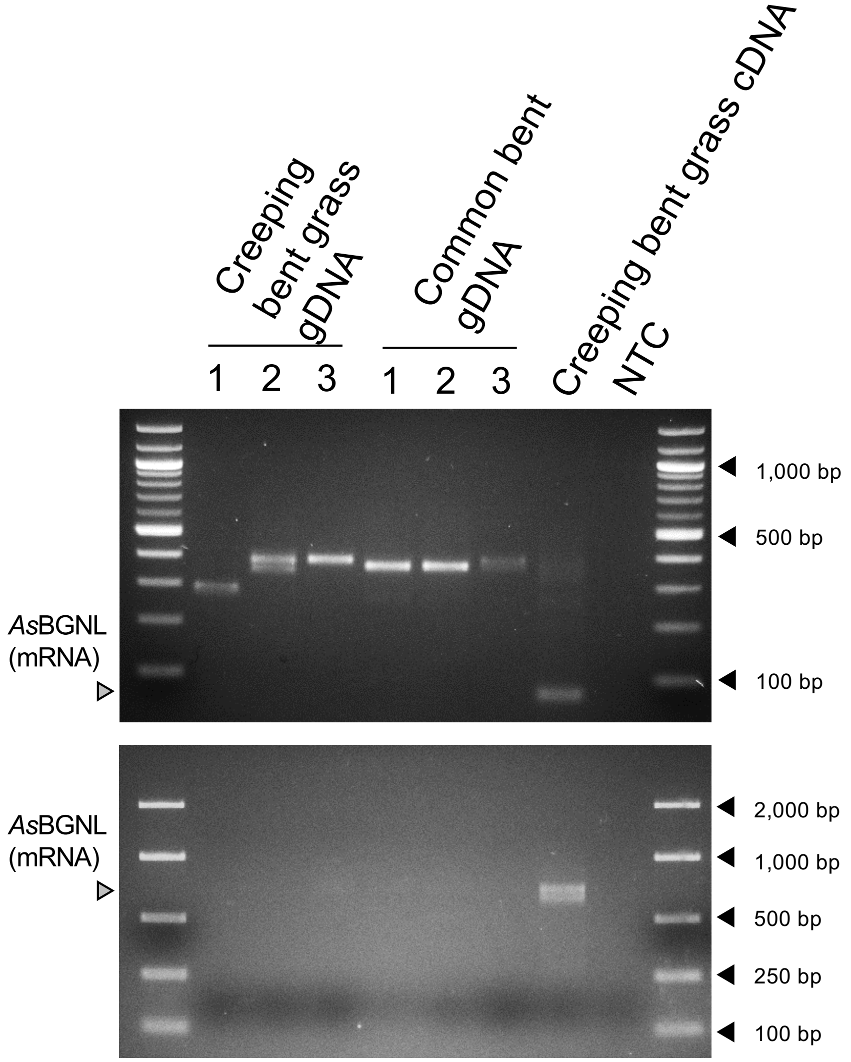

Supplement: S5 Fig — The gDNA samples of creeping bent grass and common bent grass, and cDNA sample of creeping bent grass were used as DNA template. NTC stands for ‘no template control’. PCR amplicons were visualised on an agarose gel containing the SYBR Safe stain. Based on the DNA sequence alignment result (S1 Fig), the AsBGNL_exon_F and R primers were designed. A larger fragment sizes from the gDNA template suggested presence of intron(s) in AsBGNL (upper panel). The forward primer (AsBGNL_intron_F) was designed across exon/intron boundaries of LpBGNL. The combination of the AsBGNL_intron_F and AsBGNL_R primers amplified DNA fragments from the cDNA templates, but no PCR fragments were observed from the gDNA templates, suggesting conservation of the intron position between AsBGNL and LpBGNL (lower panel). The expected positions of PCR amplicons from cDNA templates are indicated with the grey-filled arrows. The NEB 100 bp DNA Ladder (upper panel) and BIOLINE EasyLadder I (lower panel) and were used as size standard, and the sizes of representative ladders are shown with the black-filled arrows. (PDF) [file pone.0257173.s005.pdf]
