# Supplementary material for: Fungus-originated glucanase and monooxygenase genes in creeping bent grass (Agrostis stolonifera L.)
Source: PLoS One. 2021 Sep 10;16(9):e0257173. doi: 10.1371/journal.pone.0257173 (PMC8432771; doi:10.1371/journal.pone.0257173)
Supplement: S1 Table — SARDI UI denotes the unique identifier of the South Australian Research and Development Institute. (PDF) [file pone.0257173.s006.pdf]

S1 Table. Plant materials used for PCR-based screening

Common name	Scientific name	SARDI UI
Creeping bent grass	*Agrostis stolonifera*	41,545
Common bent	*Agrostis capillaris*	41,590
Annual beard grass	*Polypogon monspeliensis*	36,114
