# Supplementary material for: Fungus-originated glucanase and monooxygenase genes in creeping bent grass (Agrostis stolonifera L.)
Source: PLoS One. 2021 Sep 10;16(9):e0257173. doi: 10.1371/journal.pone.0257173 (PMC8432771; doi:10.1371/journal.pone.0257173)
Supplement: S2 Table — (PDF) [file pone.0257173.s007.pdf]

S2 Table. PCR primers designed and used in the current study

Species specifity	Traget gene	Primer name	Sequence (5'->3')	Amplicon size
Plants	*As* BGNL	AsBGNL/LpBGNL_F***	GTCGGCATGATTGAGGTTCT	178 bp (creeping bent grass)
		AsBGNL_R****	ATTGCACGTGGAGCTTGCTG	
Plants	*Lp* BGNL	AsBGNL/LpBGNL_F***	GTCGGCATGATTGAGGTTCT	178 bp (perennial ryegrass)
		LpBGNL_R	ACTGCACATGGAGCTTGTTG	
Plants/Fungi	*Lp* BGNL/ß-1,6-glucanase gene	LpBGNL_cons_F	CTGCCTCCGAGTTCGACTG	415 bp (perennial ryegrass)
		LpBGNL_cons_R	TGGATGCGCYTCGTCATCC	
Plants	*As* BGNL	AsBGNL_intron_F	ATTTCGGCGGCTGGCTCGT	690 bp (creeping bent grass/mRNA)
		AsBGNL_R****	ATTGCACGTGGAGCTTGCTG	
Plants	*As* BGNL	AsBGNL_exon_F	AGGGCATCAACAAGATCAGG	72 bp (creeping bent grass/mRNA)
		AsBGNL_exon_R	CGTCGCTCATCATCCACGGC	
Plants/fungi	*As* FMOL	AsFMOL_con_f1_AstII	GGGCGTCATCGAGCAAGTCA	174 bp (creeping bent grass)
		AsFMOL_con_r1_AstII	CACCCCTTCCACGAGTAGCAGT	
Plants	*As* FMOL	AsFMOL_ps_f1	ACAGGGTCACGCGCATAGTG	165 bp (creeping bent grass)
		AsFMOL_ps_r1	CCTTCCAGTAGTATGATCGT	
Plants	*As* FMOL	AsFMOL_SCA_f1	TCGGCGTTTCTTCTCGTAAC	N.A.
		AsFMOL_SCA_r1	ACGCATTCGTGAGATCCTTC	
Plants	*AsActin**	AsActin_F	TTGAACCCAAAAGCCAACAG	218 bp (creeping bent grass)
		AsActin_R	CCAGCAAGATCCAAACGAAG	
Plants	FT/HD3	FT-HD3_P_con_F	GAGGTGATGTGCTACGAGAG	149 bp (perennial ryegrass)
		FT-HD3_P_con_R	AGGTTGTAGAGCTCGGCRAA	
Fungi	mcf	Epichloe_mcf_F	TCGGATCATAGAAATGCCAC	205 bp (*E. festucae*)
		Epichloe_mcf_R	AAGCGATGTTTGGAAGATCT	
Fungi	ITS**	C.purpurea_D0288F	AGCCTTCTTTGCGTAGTA	98 bp**
		C.purpurea_D0289R	ACCTGATTCGAGGTCAAC	

* Reference: 13. Xiao GZ. *et. al.* (2016) Genetics and Molecular Research 15, doi:10.4238/gmr15049034

** Reference: 12. Comte, A. *et. al*. (2017) PLOS ONE 12, e0173495

*** The same forward primer was used for amplification of *As* BGNL and *Lp* BGNL-related sequences

**** The same reverse primer was used for amplification of *As* BGNL-related sequences
