# Supplementary material for: Fungus-originated glucanase and monooxygenase genes in creeping bent grass (Agrostis stolonifera L.)
Source: PLoS One. 2021 Sep 10;16(9):e0257173. doi: 10.1371/journal.pone.0257173 (PMC8432771; doi:10.1371/journal.pone.0257173)
Supplement: S3 Table — The similarity hits for AsBGNL and AsFMOL are highlighted with yellow. (PDF) [file pone.0257173.s008.pdf]

S3 Table. DNA sequence homology search result between creeping bent grass and *E. amarillans* transcriptomes

Hit UI	Query sequence (*E. amarillans* transcriptome UI)	Subject sequence (creeping bent grass transcriptome UI)	Percentage of identical matches	Alignment length	Number of mismatches	Number of gap openings	Start of alignment in query	End of alignment in query	Start of alignment in subject	End of alignment in subject	Expect value	Bit score
1	augustus_masked-contig00015-processed-gene-0.184-mRNA-1	GFQK01218355.1	83.004	253	43	0	1166	1418	275	23	1.67E-58	230
2	augustus_masked-contig00035-processed-gene-0.131-mRNA-1	GFQK01322714.1	81.585	429	61	17	454	873	2	421	1.36E-91	339
3	augustus_masked-contig00035-processed-gene-0.131-mRNA-1	GFQK01157203.1	82.719	434	73	2	42	474	433	1	1.72E-105	385
4	augustus_masked-contig00056-processed-gene-0.118-mRNA-1	GFQK01331751.1	82.073	463	71	8	52	511	58	511	9.65E-106	385
5	augustus_masked-contig00099-processed-gene-0.46-mRNA-1	GFQK01217379.1	76.455	378	76	13	1	370	858	486	7.56E-48	193
6	augustus_masked-contig00108-processed-gene-0.75-mRNA-1	GFQK01069939.1	74.219	256	64	2	301	555	1	255	9.25E-22	106
7	augustus_masked-contig00145-processed-gene-0.47-mRNA-1	GFQK01230513.1 (*As* BGNL)	93.148	1153	79	0	138	1290	1373	221	0	1692
8	augustus_masked-contig00153-processed-gene-0.66-mRNA-1	GFQK01292823.1	81.25	160	30	0	821	980	359	200	9.83E-29	130
9	augustus_masked-contig00160-processed-gene-0.42-mRNA-1	GFQK01226437.1	85.167	418	58	3	191	606	1	416	9.23E-118	425
10	augustus_masked-contig00160-processed-gene-0.42-mRNA-1	GFQK01148360.1	78.686	685	136	9	11	690	196	875	1.97E-124	448
11	augustus_masked-contig00187-processed-gene-0.75-mRNA-1	GFQK01165152.1	72.296	527	115	24	832	1344	851	1360	3.79E-30	135
12	augustus_masked-contig00187-processed-gene-0.75-mRNA-1	GFQK01167366.1	81.633	1666	277	23	94	1743	1896	244	0	1354
13	augustus_masked-contig00330-processed-gene-0.43-mRNA-1	GFQK01284743.1	79.675	615	119	6	1	612	172	783	1.38E-121	438
14	augustus_masked-contig00352-processed-gene-0.28-mRNA-1	GFQK01025519.1	77.432	257	48	7	1312	1563	1	252	8.63E-33	145
15	fgenesh_masked-contig00001-processed-gene-0.65-mRNA-1	GFQK01247828.1	85.556	360	52	0	69	428	363	4	1.37E-103	377
16	fgenesh_masked-contig00001-processed-gene-0.73-mRNA-1	GFQK01032572.1	72.666	1478	332	61	211	1655	1657	219	1.98E-117	425
17	fgenesh_masked-contig00001-processed-gene-2.139-mRNA-1	GFQK01101059.1	78.956	613	101	19	1	605	768	176	7.05E-108	392
18	fgenesh_masked-contig00002-processed-gene-2.109-mRNA-1	GFQK01035218.1	74.854	342	74	10	718	1053	6	341	3.88E-33	145
19	fgenesh_masked-contig00003-processed-gene-1.116-mRNA-1	GFQK01024857.1	85.877	701	90	8	25	721	999	304	0	737
20	fgenesh_masked-contig00006-processed-gene-0.127-mRNA-1	GFQK01053168.1	73.522	1522	348	46	280	1778	2096	607	6.34E-148	527
21	fgenesh_masked-contig00007-processed-gene-0.167-mRNA-1	GFQK01284070.1	81.226	261	47	2	2029	2288	261	2	1.11E-51	209
22	fgenesh_masked-contig00007-processed-gene-0.183-mRNA-1	GFQK01172700.1	77.821	257	55	2	411	666	2114	1859	8.59E-37	158
23	fgenesh_masked-contig00022-processed-gene-0.117-mRNA-1	GFQK01112399.1	74.865	557	101	32	1078	1613	1207	669	1.59E-54	217
24	fgenesh_masked-contig00022-processed-gene-0.117-mRNA-1	GFQK01103127.1	85.714	357	51	0	18	374	1	357	6.89E-103	377
25	fgenesh_masked-contig00022-processed-gene-0.117-mRNA-1	GFQK01135346.1	83.784	814	123	9	6	819	805	1	0	763
26	fgenesh_masked-contig00022-processed-gene-0.117-mRNA-1	GFQK01161077.1	83.694	877	139	4	1130	2004	1	875	0	824
27	fgenesh_masked-contig00048-processed-gene-0.57-mRNA-1	GFQK01195178.1	80.986	568	104	4	862	1427	1	566	3.49E-124	448
28	fgenesh_masked-contig00048-processed-gene-0.57-mRNA-1	GFQK01037719.1	82.262	778	130	8	103	876	774	1	0	665
29	fgenesh_masked-contig00060-processed-gene-0.57-mRNA-1	GFQK01158934.1	80.556	180	31	4	153	330	453	276	1.01E-30	135
30	fgenesh_masked-contig00060-processed-gene-0.68-mRNA-1	GFQK01067904.1	87.179	468	54	6	73	537	166	630	1.71E-148	527
31	fgenesh_masked-contig00067-processed-gene-0.95-mRNA-1	GFQK01156802.1	88.119	303	34	2	68	369	302	1	5.96E-98	359
32	fgenesh_masked-contig00100-processed-gene-0.65-mRNA-1	GFQK01079213.1	80.634	284	55	0	22	305	403	120	1.69E-56	220
33	fgenesh_masked-contig00100-processed-gene-0.65-mRNA-1	GFQK01023187.1	89.744	312	32	0	1	312	486	175	2.02E-110	399
34	fgenesh_masked-contig00109-processed-gene-0.50-mRNA-1	GFQK01149322.1	84.585	506	74	4	24	527	854	351	3.81E-140	499
35	fgenesh_masked-contig00169-processed-gene-0.28-mRNA-1	GFQK01222877.1	78.627	510	105	4	324	831	2516	2009	1.62E-90	335
36	fgenesh_masked-contig00169-processed-gene-0.28-mRNA-1	GFQK01201387.1	82.411	506	81	8	329	830	680	179	1.56E-120	435
37	fgenesh_masked-contig00192-processed-gene-0.40-mRNA-1	GFQK01284855.1	76.049	405	87	8	156	555	1	400	1.18E-50	202
38	fgenesh_masked-contig00230-processed-gene-0.30-mRNA-1	GFQK01095557.1	82.877	584	90	9	1441	2019	962	384	1.35E-144	516
39	fgenesh_masked-contig00237-processed-gene-0.48-mRNA-1	GFQK01114691.1 (AsFMOL)	95.455	660	24	4	1	660	1488	835	0	1048
40	fgenesh_masked-contig00237-processed-gene-0.48-mRNA-1	GFQK01114691.1 (AsFMOL)	95.553	787	20	1	660	1446	787	16	0	1245
41	fgenesh_masked-contig00276-processed-gene-0.19-mRNA-1	GFQK01023187.1	84.936	312	47	0	1	312	486	175	2.10E-85	316
42	fgenesh_masked-contig00330-processed-gene-0.27-mRNA-1	GFQK01090050.1	90	200	20	0	22	221	200	1	7.38E-68	259
43	fgenesh_masked-contig00375-processed-gene-0.19-mRNA-1	GFQK01329347.1	81.728	706	123	6	1	703	135	837	2.08E-165	584
44	fgenesh_masked-contig00378-processed-gene-0.24-mRNA-1	GFQK01276716.1	76.408	373	74	12	217	582	311	676	9.27E-47	189
45	fgenesh_masked-contig00440-processed-gene-0.23-mRNA-1	GFQK01234327.1	82.292	384	66	2	925	1307	386	4	5.77E-89	331
46	fgenesh_masked-contig00527-processed-gene-0.15-mRNA-1	GFQK01283283.1	84.298	242	36	2	667	907	241	1	2.29E-60	235
47	fgenesh_masked-contig00527-processed-gene-0.15-mRNA-1	GFQK01262810.1	89.451	237	23	2	688	923	2	237	2.88E-79	298
48	fgenesh_masked-contig00527-processed-gene-0.15-mRNA-1	GFQK01198822.1	83.495	412	62	6	1	409	409	1	1.00E-103	379
49	fgenesh_masked-contig00613-processed-gene-0.9-mRNA-1	GFQK01009811.1	81.675	191	31	4	19	207	1210	1022	3.08E-37	156
50	fgenesh_masked-contig00703-processed-gene-0.13-mRNA-1	GFQK01160051.1	73.467	848	210	13	196	1034	895	54	3.31E-81	305

S3 Table. (Cont'd)

Hit UI	Query sequence (*E. amarillans* transcriptome UI)	Subject sequence (creeping bent grass transcriptome UI)	Percentage of identical matches	Alignment length	Number of mismatch es	Number of gap openings	Start of alignment in query	End of alignment in query	Start of alignment in subject	End of alignment in subject	Expect value	Bit score
51	fgenesh_masked-contig00944-processed-gene-0.6-mRNA-1	GFQK01294866.1	78.107	507	100	8	469	965	723	218	3.38E-83	311
52	genemark-contig00001-processed-gene-2.49-mRNA-1	GFQK01332812.1	81.333	150	26	2	403	551	1386	1238	9.44E-26	121
53	genemark-contig00022-processed-gene-0.28-mRNA-1	GFQK01257057.1	86.111	144	17	3	93	234	472	330	9.54E-36	152
54	genemark-contig00211-processed-gene-0.2-mRNA-1	GFQK01047543.1	84.146	246	37	2	2089	2333	245	1	1.84E-60	237
55	genemark-contig00211-processed-gene-0.2-mRNA-1	GFQK01328148.1	84.932	365	55	0	621	985	2730	2366	1.74E-100	370
56	genemark-contig00211-processed-gene-0.2-mRNA-1	GFQK01328148.1	81.082	1940	333	29	1239	3161	2115	193	0	1519
57	genemark-contig00230-processed-gene-0.4-mRNA-1	GFQK01157225.1	76.381	724	152	13	1	722	117	823	1.12E-101	372
58	genemark-contig00678-processed-gene-0.2-mRNA-1	GFQK01302511.1	81.818	462	74	7	2176	2629	369	828	2.43E-103	379
59	maker-contig00001-augustus-gene-2.340-mRNA-1	GFQK01174630.1	84.964	419	57	5	277	692	1	416	3.99E-116	420
60	maker-contig00001-augustus-gene-2.340-mRNA-2	GFQK01174630.1	84.964	419	57	5	238	653	1	416	3.78E-116	420
61	maker-contig00001-augustus-gene-2.343-mRNA-1	GFQK01303847.1	75.394	508	117	8	145	648	507	4	2.16E-61	239
62	maker-contig00001-augustus-gene-2.343-mRNA-1	GFQK01308429.1	86.667	570	76	0	63	632	1	570	8.47E-180	632
63	maker-contig00002-fgenesh-gene-1.161-mRNA-1	GFQK01230902.1	85.217	115	17	0	1011	1125	232	118	2.62E-25	119
64	maker-contig00002-fgenesh-gene-2.160-mRNA-1	GFQK01291390.1	77.358	530	108	11	1119	1642	1050	527	1.15E-80	303
65	maker-contig00003-snap-gene-1.309-mRNA-1	GFQK01109945.1	79.07	344	70	2	3252	3594	289	631	9.47E-60	235
66	maker-contig00010-fgenesh-gene-0.222-mRNA-1	GFQK01112822.1	80.201	596	102	14	538	1125	594	7	1.52E-119	433
67	maker-contig00011-augustus-gene-0.310-mRNA-1	GFQK01169380.1	81.731	208	36	2	1322	1528	207	1	4.49E-41	172
68	maker-contig00011-fgenesh-gene-0.225-mRNA-1	GFQK01060504.1	79.84	625	122	4	1	623	532	1154	5.62E-126	453
69	maker-contig00012-fgenesh-gene-0.198-mRNA-1	GFQK01308193.1	80.872	298	47	10	2116	2408	294	2	4.03E-57	226
70	maker-contig00013-augustus-gene-0.327-mRNA-1	GFQK01086737.1	87.117	163	21	0	32	194	127	289	1.21E-45	185
71	maker-contig00013-augustus-gene-0.327-mRNA-1	GFQK01152023.1	88.172	186	20	2	79	263	186	2	3.32E-56	220
72	maker-contig00013-augustus-gene-0.327-mRNA-1	GFQK01096195.1	81.311	412	69	7	160	567	467	60	1.90E-88	327
73	maker-contig00013-augustus-gene-0.327-mRNA-1	GFQK01124140.1	86.435	317	41	2	267	582	1	316	5.26E-94	346
74	maker-contig00013-augustus-gene-0.327-mRNA-2	GFQK01086737.1	87.117	163	21	0	104	266	127	289	1.37E-45	185
75	maker-contig00013-augustus-gene-0.327-mRNA-2	GFQK01152023.1	88.172	186	20	2	151	335	186	2	3.75E-56	220
76	maker-contig00013-augustus-gene-0.327-mRNA-2	GFQK01096195.1	81.311	412	69	7	232	639	467	60	2.15E-88	327
77	maker-contig00013-augustus-gene-0.327-mRNA-2	GFQK01124140.1	86.435	317	41	2	339	654	1	316	5.93E-94	346
78	maker-contig00014-snap-gene-0.338-mRNA-1	GFQK01227634.1	73.664	786	193	10	964	1742	34	812	2.11E-77	292
79	maker-contig00015-augustus-gene-0.291-mRNA-1	GFQK01282059.1	84.689	209	32	0	41	249	209	1	5.47E-53	209
80	maker-contig00015-augustus-gene-0.291-mRNA-2	GFQK01282059.1	86.508	252	34	0	1	252	252	1	1.49E-73	278
81	maker-contig00016-fgenesh-gene-0.240-mRNA-1	GFQK01031285.1	83.333	336	56	0	22	357	336	1	2.88E-83	311
82	maker-contig00016-fgenesh-gene-0.246-mRNA-1	GFQK01294982.1	80.493	1502	279	11	80	1574	1705	211	0	1138
83	maker-contig00018-fgenesh-gene-0.257-mRNA-1	GFQK01144246.1	79.343	426	69	16	785	1202	419	5	4.36E-74	281
84	maker-contig00020-fgenesh-gene-0.198-mRNA-1	GFQK01318997.1	79.518	249	45	4	304	549	566	321	1.07E-41	172
85	maker-contig00022-augustus-gene-0.256-mRNA-1	GFQK01323720.1	90.347	259	25	0	72	330	508	250	1.43E-92	340
86	maker-contig00022-fgenesh-gene-0.190-mRNA-1	GFQK01081120.1	78.83	855	161	16	109	956	1043	202	1.07E-157	558
87	maker-contig00023-fgenesh-gene-0.170-mRNA-1	GFQK01332864.1	89.076	119	13	0	935	1053	269	151	2.91E-34	148
88	maker-contig00023-fgenesh-gene-0.180-mRNA-1	GFQK01330017.1	88.776	98	11	0	64	161	98	1	6.73E-26	121
89	maker-contig00026-fgenesh-gene-0.170-mRNA-1	GFQK01236250.1	80.186	429	85	0	151	579	691	1119	2.12E-86	322
90	maker-contig00028-fgenesh-gene-0.131-mRNA-1	GFQK01163683.1	78.947	209	44	0	130	338	957	749	1.66E-32	143
91	maker-contig00028-fgenesh-gene-0.132-mRNA-1	GFQK01165555.1	88	150	18	0	1	150	150	1	1.38E-43	178
92	maker-contig00030-fgenesh-gene-0.184-mRNA-1	GFQK01011688.1	71.971	842	210	20	215	1046	240	1065	5.15E-57	224
93	maker-contig00033-fgenesh-gene-0.168-mRNA-1	GFQK01266058.1	81.513	1044	177	14	94	1129	1273	238	0	845
94	maker-contig00034-augustus-gene-0.220-mRNA-1	GFQK01271195.1	75.244	513	109	17	580	1083	1	504	3.73E-58	228
95	maker-contig00034-augustus-gene-0.220-mRNA-1	GFQK01101425.1	81.863	1009	179	4	1	1007	1009	3	0	846
96	maker-contig00034-augustus-gene-0.220-mRNA-2	GFQK01271195.1	75.244	513	109	17	826	1329	1	504	4.59E-58	228
97	maker-contig00034-augustus-gene-0.220-mRNA-2	GFQK01101425.1	81.562	960	173	4	296	1253	960	3	0	789
98	maker-contig00040-fgenesh-gene-0.186-mRNA-1	GFQK01089002.1	83.81	210	32	2	628	836	2	210	5.38E-49	198
99	maker-contig00044-fgenesh-gene-0.148-mRNA-1	GFQK01196040.1	77.519	774	162	10	151	918	220	987	1.40E-126	455
100	maker-contig00045-fgenesh-gene-0.124-mRNA-1	GFQK01291577.1	85.401	137	20	0	526	662	138	2	5.04E-32	143

S3 Table. (Cont'd)

Hit UI	Query sequence (*E. amarillans* transcriptome UI)	Subject sequence (creeping bent grass transcriptome UI)	Percentage of identical matches	Alignment length	Number of mismatch es	Number of gap openings	Start of alignment in query	End of alignment in query	Start of alignment in subject	End of alignment in subject	Expect value	Bit score
101	maker-contig00045-fgenesh-gene-0.124-mRNA-1	GFQK01154740.1	78.799	1849	382	6	118	1961	342	2185	0	1234
102	maker-contig00051-fgenesh-gene-0.153-mRNA-1	GFQK01247547.1	86.598	194	26	0	205	398	195	2	1.85E-54	215
103	maker-contig00051-fgenesh-gene-0.153-mRNA-1	GFQK01117741.1	81.658	398	61	11	220	611	393	2	3.82E-86	320
104	maker-contig00052-fgenesh-gene-0.107-mRNA-1	GFQK01072672.1	78.781	1329	259	20	1	1322	1490	178	0	870
105	maker-contig00058-augustus-gene-0.216-mRNA-1	GFQK01008056.1	82.164	499	83	6	11	506	251	746	2.22E-117	424
106	maker-contig00062-snap-gene-0.199-mRNA-1	GFQK01140289.1	81.573	445	74	8	22	462	441	1	6.24E-98	361
107	maker-contig00062-snap-gene-0.199-mRNA-1	GFQK01151842.1	79.329	566	113	4	1339	1902	887	324	6.15E-108	394
108	maker-contig00062-snap-gene-0.199-mRNA-1	GFQK01046790.1	82.609	460	74	5	7	463	457	1	3.67E-110	401
109	maker-contig00062-snap-gene-0.199-mRNA-1	GFQK01108294.1	83.306	605	101	0	529	1133	605	1	2.06E-157	558
110	maker-contig00063-fgenesh-gene-0.115-mRNA-1	GFQK01310953.1	77.803	446	91	7	201	642	245	686	1.28E-70	268
111	maker-contig00063-fgenesh-gene-0.115-mRNA-1	GFQK01225466.1	89.623	318	33	0	270	587	1	318	9.37E-112	405
112	maker-contig00063-fgenesh-gene-0.121-mRNA-1	GFQK01112899.1	89.216	102	11	0	847	948	1	102	3.39E-28	128
113	maker-contig00063-fgenesh-gene-0.121-mRNA-1	GFQK01022953.1	82.71	428	74	0	29	456	428	1	2.36E-104	381
114	maker-contig00063-fgenesh-gene-0.121-mRNA-1	GFQK01133538.1	83.784	407	66	0	439	845	1	407	5.07E-106	387
115	maker-contig00063-fgenesh-gene-0.121-mRNA-1	GFQK01054246.1	77.594	906	187	13	51	948	127	1024	1.71E-150	534
116	maker-contig00065-augustus-gene-0.165-mRNA-1	GFQK01268304.1	85.714	147	21	0	1	147	86	232	7.93E-37	156
117	maker-contig00066-snap-gene-0.201-mRNA-1	GFQK01327446.1	85.808	613	85	2	1	612	781	170	0	649
118	maker-contig00067-augustus-gene-0.199-mRNA-1	GFQK01231738.1	80.606	330	62	2	733	1061	329	1	9.63E-66	254
119	maker-contig00067-augustus-gene-0.199-mRNA-1	GFQK01183058.1	84.698	281	40	2	124	401	784	504	5.72E-73	278
120	maker-contig00067-augustus-gene-0.199-mRNA-1	GFQK01099594.1	82.283	1016	164	14	436	1443	2800	3807	0	865
121	maker-contig00067-fgenesh-gene-0.130-mRNA-1	GFQK01172231.1	76.414	1467	316	22	133	1587	1578	130	0	765
122	maker-contig00067-fgenesh-gene-0.135-mRNA-1	GFQK01278983.1	84.06	596	92	3	15	608	121	715	8.82E-162	571
123	maker-contig00072-fgenesh-gene-0.143-mRNA-1	GFQK01302297.1	75.191	262	65	0	20	281	773	512	6.30E-27	124
124	maker-contig00072-fgenesh-gene-0.143-mRNA-1	GFQK01061937.1	75.064	389	78	15	322	702	1244	867	1.34E-38	163
125	maker-contig00072-fgenesh-gene-0.143-mRNA-1	GFQK01285647.1	79.693	261	47	5	1	258	60	317	1.02E-44	183
126	maker-contig00072-fgenesh-gene-0.143-mRNA-1	GFQK01084468.1	82.405	341	60	0	895	1235	1	341	3.51E-79	298
127	maker-contig00072-fgenesh-gene-0.143-mRNA-1	GFQK01019572.1	75.445	1067	225	29	55	1104	1382	336	2.51E-135	484
128	maker-contig00072-fgenesh-gene-0.143-mRNA-1	GFQK01206604.1	76.434	1116	223	29	24	1119	106	1201	2.42E-160	568
129	maker-contig00077-augustus-gene-0.188-mRNA-1	GFQK01094026.1	72.537	1147	293	18	367	1502	255	1390	8.54E-96	353
130	maker-contig00077-augustus-gene-0.188-mRNA-1	GFQK01007318.1	78.637	763	154	9	750	1507	1	759	3.73E-139	497
131	maker-contig00077-fgenesh-gene-0.130-mRNA-1	GFQK01208464.1	82.468	462	78	2	1	459	637	176	8.52E-111	401
132	maker-contig00077-fgenesh-gene-0.148-mRNA-1	GFQK01197190.1	84.488	303	45	2	169	470	318	17	2.09E-79	298
133	maker-contig00078-fgenesh-gene-0.117-mRNA-1	GFQK01125825.1	80.496	282	53	2	78	358	586	306	1.06E-54	215
134	maker-contig00081-snap-gene-0.150-mRNA-1	GFQK01201656.1	77.301	815	173	10	1	809	1126	318	5.97E-131	470
135	maker-contig00082-fgenesh-gene-0.75-mRNA-1	GFQK01005361.1	85.567	97	14	0	339	435	40	136	9.14E-21	102
136	maker-contig00082-snap-gene-0.111-mRNA-1	GFQK01268369.1	79.304	517	102	3	11	526	122	634	2.53E-97	357
137	maker-contig00082-snap-gene-0.111-mRNA-1	GFQK01003168.1	80.536	560	97	9	6	559	912	359	3.18E-116	420
138	maker-contig00085-fgenesh-gene-0.108-mRNA-1	GFQK01257596.1	85.302	381	54	2	1222	1601	381	2	2.31E-107	392
139	maker-contig00085-snap-gene-0.157-mRNA-1	GFQK01143232.1	79.503	1449	264	28	1	1434	1643	213	0	1000
140	maker-contig00087-fgenesh-gene-0.122-mRNA-1	GFQK01227926.1	77.143	1260	276	12	1	1254	1867	614	0	721
141	maker-contig00092-augustus-gene-0.144-mRNA-1	GFQK01131639.1	86.321	212	26	2	13	224	50	258	4.80E-58	228
142	maker-contig00092-augustus-gene-0.144-mRNA-1	GFQK01148542.1	77.672	421	72	18	232	638	737	325	7.97E-61	237
143	maker-contig00092-augustus-gene-0.144-mRNA-1	GFQK01068442.1	77.846	650	120	18	1	638	57	694	3.48E-104	381
144	maker-contig00092-augustus-gene-0.144-mRNA-1	GFQK01247326.1	84.543	427	57	7	537	960	495	75	3.43E-114	414
145	maker-contig00092-augustus-gene-0.144-mRNA-1	GFQK01182259.1	85.903	454	61	2	933	1386	1	451	3.33E-134	481
146	maker-contig00092-augustus-gene-0.144-mRNA-1	GFQK01060217.1	83.663	1365	212	10	13	1373	308	1665	0	1275
147	maker-contig00100-fgenesh-gene-0.98-mRNA-1	GFQK01154430.1	81.944	144	22	4	324	465	143	2	1.54E-25	119
148	maker-contig00101-fgenesh-gene-0.90-mRNA-1	GFQK01234205.1	74.1	722	138	31	1	705	143	832	1.43E-65	252
149	maker-contig00102-fgenesh-gene-0.40-mRNA-1	GFQK01029076.1	77.159	359	77	5	500	856	1	356	3.29E-50	204
150	maker-contig00102-fgenesh-gene-0.40-mRNA-1	GFQK01093605.1	80.915	503	90	6	1807	2306	98	597	6.54E-107	392

S3 Table. (Cont'd)

Hit UI	Query sequence (*E. amarillans* transcriptome UI)	Subject sequence (creeping bent grass transcriptome UI)	Percentage of identical matches	Alignment length	Number of mismatches	Number of gap openings	Start of alignment in query	End of alignment in query	Start of alignment in subject	End of alignment in subject	Expect value	Bit score
151	maker-contig00103-snap-gene-0.157-mRNA-1	GFQK01070007.1	80.62	258	44	6	84	338	260	6	3.47E-48	195
152	maker-contig00103-snap-gene-0.157-mRNA-1	GFQK01207449.1	83.592	451	72	2	16	465	455	6	1.46E-116	422
153	maker-contig00113-fgenesh-gene-0.112-mRNA-1	GFQK01021932.1	86.029	136	19	0	342	477	1	136	5.48E-34	147
154	maker-contig00113-fgenesh-gene-0.112-mRNA-1	GFQK01158418.1	81.348	445	81	2	37	480	71	514	1.80E-98	361
155	maker-contig00116-fgenesh-gene-0.97-mRNA-1	GFQK01129111.1	83.186	226	38	0	1199	1424	228	3	7.02E-52	207
156	maker-contig00116-fgenesh-gene-0.97-mRNA-1	GFQK01031134.1	83.394	277	44	2	1273	1548	402	127	2.47E-66	255
157	maker-contig00116-fgenesh-gene-0.97-mRNA-1	GFQK01324239.1	76.03	534	117	10	157	683	142	671	1.14E-69	267
158	maker-contig00116-fgenesh-gene-0.97-mRNA-1	GFQK01104912.1	91.827	208	17	0	944	1151	210	3	6.78E-77	291
159	maker-contig00116-fgenesh-gene-0.97-mRNA-1	GFQK01163948.1	89.535	258	27	0	616	873	1	258	5.16E-88	327
160	maker-contig00116-fgenesh-gene-0.97-mRNA-1	GFQK01247182.1	83.278	598	98	2	679	1275	597	1	1.01E-154	549
161	maker-contig00116-fgenesh-gene-0.97-mRNA-1	GFQK01248910.1	80.945	1249	224	13	247	1488	1604	363	0	976
162	maker-contig00122-fgenesh-gene-0.77-mRNA-1	GFQK01067486.1	76	275	52	14	12	279	89	356	5.98E-29	130
163	maker-contig00122-fgenesh-gene-0.77-mRNA-1	GFQK01009738.1	80.549	437	79	4	161	594	616	183	1.53E-89	331
164	maker-contig00123-fgenesh-gene-0.66-mRNA-1	GFQK01097968.1	77.016	248	55	2	86	332	247	1	4.48E-32	141
165	maker-contig00127-augustus-gene-0.141-mRNA-1	GFQK01027841.1	81.402	656	114	6	242	893	975	324	8.65E-149	529
166	maker-contig00127-augustus-gene-0.141-mRNA-2	GFQK01027841.1	81.402	656	114	6	197	848	975	324	8.27E-149	529
167	maker-contig00134-fgenesh-gene-0.73-mRNA-1	GFQK01158250.1	84.066	364	55	3	454	816	382	21	3.43E-94	348
168	maker-contig00134-fgenesh-gene-0.73-mRNA-1	GFQK01214042.1	85.278	360	53	0	826	1185	14	373	2.03E-101	372
169	maker-contig00141-fgenesh-gene-0.79-mRNA-1	GFQK01046280.1	78.4	375	79	2	15	388	376	3	5.77E-63	243
170	maker-contig00141-fgenesh-gene-0.83-mRNA-1	GFQK01257478.1	81.25	304	55	2	297	599	304	2	2.66E-63	244
171	maker-contig00141-fgenesh-gene-0.83-mRNA-1	GFQK01325394.1	84.027	745	117	2	19	762	950	207	0	715
172	maker-contig00153-fgenesh-gene-0.75-mRNA-1	GFQK01034497.1	78.4	250	47	6	875	1121	247	2	7.46E-36	156
173	maker-contig00155-fgenesh-gene-0.88-mRNA-1	GFQK01310744.1	75.742	1348	317	10	229	1571	1559	217	0	669
174	maker-contig00159-fgenesh-gene-0.100-mRNA-1	GFQK01120088.1	77.87	1934	380	40	1	1910	100	2009	0	1157
175	maker-contig00159-fgenesh-gene-0.99-mRNA-1	GFQK01234786.1	86.735	588	75	1	45	629	1165	578	0	651
176	maker-contig00163-fgenesh-gene-0.72-mRNA-1	GFQK01024348.1	81.277	235	40	4	1	233	316	84	1.50E-45	187
177	maker-contig00163-fgenesh-gene-0.72-mRNA-1	GFQK01275355.1	77.676	327	69	4	306	630	325	1	2.49E-48	196
178	maker-contig00163-fgenesh-gene-0.72-mRNA-1	GFQK01014298.1	83.478	230	34	3	749	976	1	228	8.89E-53	211
179	maker-contig00163-fgenesh-gene-0.72-mRNA-1	GFQK01221552.1	83.662	557	81	7	1201	1752	552	1	1.68E-144	516
180	maker-contig00163-fgenesh-gene-0.72-mRNA-1	GFQK01301858.1	75.136	1476	323	38	961	2414	1626	173	0	652
181	maker-contig00163-fgenesh-gene-0.72-mRNA-1	GFQK01100699.1	82.847	857	143	4	352	1206	855	1	0	765
182	maker-contig00163-fgenesh-gene-0.72-mRNA-1	GFQK01043864.1	82.913	913	141	14	303	1206	907	1	0	808
183	maker-contig00163-fgenesh-gene-0.72-mRNA-1	GFQK01278770.1	76.731	2441	495	58	1	2406	142	2544	0	1293
184	maker-contig00164-fgenesh-gene-0.83-mRNA-1	GFQK01186248.1	79.186	1105	220	9	241	1340	1149	50	0	758
185	maker-contig00169-fgenesh-gene-0.58-mRNA-1	GFQK01289185.1	79.795	391	73	5	64	451	210	597	4.11E-74	279
186	maker-contig00169-fgenesh-gene-0.58-mRNA-1	GFQK01260419.1	81.941	371	55	9	79	443	424	60	2.44E-81	303
187	maker-contig00187-fgenesh-gene-0.88-mRNA-1	GFQK01293354.1	78.758	306	61	4	99	402	304	1	2.11E-50	202
188	maker-contig00187-fgenesh-gene-0.88-mRNA-1	GFQK01045708.1	81.329	316	43	9	337	644	8	315	1.24E-62	243
189	maker-contig00187-fgenesh-gene-0.88-mRNA-1	GFQK01266869.1	82.322	956	159	10	6	956	951	1	0	821
190	maker-contig00187-snap-gene-0.140-mRNA-1	GFQK01038219.1	71.532	829	206	27	166	979	3465	2652	1.37E-48	196
191	maker-contig00200-snap-gene-0.101-mRNA-1	GFQK01019932.1	79.876	969	190	3	145	1109	1136	169	0	704
192	maker-contig00203-fgenesh-gene-0.38-mRNA-1	GFQK01034508.1	79.926	538	106	2	13	549	538	2	2.40E-108	394
193	maker-contig00217-fgenesh-gene-0.87-mRNA-1	GFQK01203308.1	83.237	173	27	2	18	189	4	175	1.99E-37	158
194	maker-contig00220-fgenesh-gene-0.58-mRNA-1	GFQK01273943.1	74.97	831	194	12	133	956	1133	310	6.37E-101	370
195	maker-contig00222-snap-gene-0.108-mRNA-1	GFQK01107101.1	76.11	766	173	10	460	1220	846	86	1.52E-107	392
196	maker-contig00222-snap-gene-0.108-mRNA-1	GFQK01142635.1	78.125	800	171	4	435	1232	333	1130	1.87E-141	505
197	maker-contig00222-snap-gene-0.108-mRNA-1	GFQK01025736.1	77.986	854	172	16	376	1221	892	47	1.86E-146	521
198	maker-contig00222-snap-gene-0.108-mRNA-1	GFQK01068297.1	85.38	513	75	0	726	1238	647	135	8.57E-150	532
199	maker-contig00224-snap-gene-0.113-mRNA-1	GFQK01159330.1	72.041	490	121	16	886	1367	791	1272	4.28E-29	132
200	maker-contig00232-fgenesh-gene-0.63-mRNA-1	GFQK01068900.1	76.485	421	75	19	1209	1613	490	78	7.28E-52	207

S3 Table. (Cont'd)

Hit UI	Query sequence (*E. amarillans* transcriptome UI)	Subject sequence (creeping bent grass transcriptome UI)	Percentage of identical matches	Alignment length	Number of mismatch es	Number of gap openings	Start of alignment in query	End of alignment in query	Start of alignment in subject	End of alignment in subject	Expect value	Bit score
201	maker-contig00239-fgenesh-gene-0.68-mRNA-1	GFQK01183355.1	74.021	843	199	18	460	1292	5	837	2.66E-87	326
202	maker-contig00246-fgenesh-gene-0.46-mRNA-1	GFQK01119422.1	74.26	1352	305	34	416	1746	436	1765	1.73E-148	529
203	maker-contig00251-fgenesh-gene-0.62-mRNA-1	GFQK01128747.1	79.021	1349	269	13	73	1414	1448	107	0	911
204	maker-contig00258-fgenesh-gene-0.62-mRNA-1	GFQK01108502.1	80.282	355	59	9	1659	2006	451	101	1.04E-66	257
205	maker-contig00262-fgenesh-gene-0.38-mRNA-1	GFQK01184408.1	82.085	1055	183	6	1	1052	397	1448	0	896
206	maker-contig00276-fgenesh-gene-0.36-mRNA-1	GFQK01048047.1	87.179	117	15	0	1	117	117	1	3.05E-30	134
207	maker-contig00276-fgenesh-gene-0.36-mRNA-1	GFQK01209420.1	78.079	406	87	2	1	405	9	413	6.24E-67	255
208	maker-contig00278-snap-gene-0.93-mRNA-1	GFQK01046068.1	70.991	817	212	19	315	1122	801	1	2.57E-41	172
209	maker-contig00296-fgenesh-gene-0.40-mRNA-1	GFQK01225239.1	81.137	387	67	5	375	758	1	384	1.21E-81	305
210	maker-contig00296-fgenesh-gene-0.40-mRNA-1	GFQK01221473.1	86.477	281	36	2	114	393	280	1	3.37E-82	307
211	maker-contig00305-fgenesh-gene-0.46-mRNA-1	GFQK01214545.1	82.292	288	51	0	328	615	288	1	2.08E-64	250
212	maker-contig00305-fgenesh-gene-0.46-mRNA-1	GFQK01143421.1	84.329	1557	230	14	622	2171	1551	2	0	1511
213	maker-contig00305-fgenesh-gene-0.50-mRNA-1	GFQK01041026.1	81.458	1289	225	11	99	1380	1354	73	0	1044
214	maker-contig00331-augustus-gene-0.80-mRNA-1	GFQK01182190.1	83.502	297	49	0	345	641	1	297	2.16E-73	278
215	maker-contig00331-snap-gene-0.85-mRNA-1	GFQK01062597.1	83.115	764	123	4	1	761	32	792	0	691
216	maker-contig00335-fgenesh-gene-0.45-mRNA-1	GFQK01327946.1	83.179	541	89	2	1	540	144	683	1.77E-138	494
217	maker-contig00335-fgenesh-gene-0.45-mRNA-1	GFQK01215092.1	84.112	535	83	2	1	534	534	1	3.79E-145	516
218	maker-contig00344-augustus-gene-0.63-mRNA-1	GFQK01091261.1	77.121	660	137	13	1786	2438	67	719	1.39E-100	370
219	maker-contig00352-fgenesh-gene-0.35-mRNA-1	GFQK01096787.1	75.771	227	51	4	67	291	530	306	1.01E-23	111
220	maker-contig00367-fgenesh-gene-0.20-mRNA-1	GFQK01318890.1	79.429	175	34	2	652	825	667	840	3.19E-26	122
221	maker-contig00367-fgenesh-gene-0.20-mRNA-1	GFQK01204684.1	79.784	371	65	10	381	746	366	1	6.48E-68	261
222	maker-contig00367-fgenesh-gene-0.20-mRNA-1	GFQK01047794.1	80.388	464	79	1	751	1214	453	2	2.25E-92	342
223	maker-contig00372-augustus-gene-0.58-mRNA-1	GFQK01197445.1	74.649	1424	342	18	3219	4631	1859	444	4.42E-173	612
224	maker-contig00383-fgenesh-gene-0.48-mRNA-1	GFQK01273689.1	82.917	1042	174	4	31	1070	1372	333	0	935
225	maker-contig00393-augustus-gene-0.17-mRNA-1	GFQK01167742.1	78.065	1044	214	12	553	1590	434	1468	0	645
226	maker-contig00403-fgenesh-gene-0.39-mRNA-1	GFQK01310175.1	83.085	201	34	0	208	408	555	355	3.15E-45	183
227	maker-contig00404-fgenesh-gene-0.40-mRNA-1	GFQK01226761.1	72	725	181	19	271	984	3222	2509	4.31E-48	195
228	maker-contig00407-fgenesh-gene-0.24-mRNA-1	GFQK01089757.1	80.149	403	77	1	783	1185	819	420	3.07E-79	298
229	maker-contig00407-fgenesh-gene-0.24-mRNA-1	GFQK01169952.1	80.301	797	151	2	38	831	103	896	2.70E-169	597
230	maker-contig00408-fgenesh-gene-0.37-mRNA-1	GFQK01125600.1	82.336	351	54	6	28	374	474	128	9.27E-80	298
231	maker-contig00414-fgenesh-gene-0.30-mRNA-1	GFQK01165956.1	78.934	1201	235	16	234	1425	1487	296	0	800
232	maker-contig00418-augustus-gene-0.42-mRNA-1	GFQK01034265.1	83.065	248	41	1	776	1022	1468	1715	5.45E-57	224
233	maker-contig00440-augustus-gene-0.45-mRNA-1	GFQK01286359.1	80.315	381	70	4	39	417	412	35	1.16E-74	283
234	maker-contig00454-fgenesh-gene-0.18-mRNA-1	GFQK01284084.1	86.316	95	13	0	566	660	471	565	4.13E-21	104
235	maker-contig00476-fgenesh-gene-0.24-mRNA-1	GFQK01244640.1	84.278	388	57	3	33	418	537	152	4.70E-103	375
236	maker-contig00484-fgenesh-gene-0.44-mRNA-1	GFQK01133256.1	85.117	383	55	2	10	391	516	135	1.56E-107	390
237	maker-contig00484-fgenesh-gene-0.45-mRNA-1	GFQK01170798.1	78.853	279	53	6	913	1188	12	287	9.04E-45	183
238	maker-contig00484-fgenesh-gene-0.45-mRNA-1	GFQK01192303.1	90	280	26	2	916	1194	719	441	3.89E-98	361
239	maker-contig00484-fgenesh-gene-0.45-mRNA-1	GFQK01218475.1	80.887	654	114	7	14	663	647	1	1.70E-141	505
240	maker-contig00484-fgenesh-gene-0.45-mRNA-1	GFQK01078130.1	87.5	800	100	0	10	809	227	1026	0	924
241	maker-contig00484-fgenesh-gene-0.46-mRNA-1	GFQK01066502.1	83.663	404	60	3	37	434	695	292	4.99E-103	375
242	maker-contig00503-fgenesh-gene-0.32-mRNA-1	GFQK01159858.1	83.659	410	67	0	754	1163	545	136	6.32E-106	387
243	maker-contig00503-fgenesh-gene-0.32-mRNA-1	GFQK01077321.1	86.189	391	52	2	385	774	1	390	1.73E-116	422
244	maker-contig00503-fgenesh-gene-0.32-mRNA-1	GFQK01292397.1	75.913	1150	245	27	25	1158	2	1135	3.52E-158	560
245	maker-contig00544-fgenesh-gene-0.24-mRNA-1	GFQK01330861.1	77.778	252	44	10	267	512	298	543	1.88E-33	145
246	maker-contig00552-fgenesh-gene-0.20-mRNA-1	GFQK01328502.1	77.688	372	71	12	314	679	3	368	8.13E-55	217
247	maker-contig00603-augustus-gene-0.20-mRNA-1	GFQK01236296.1	75.397	378	79	12	527	897	1255	885	9.84E-41	171
248	maker-contig00678-fgenesh-gene-0.19-mRNA-1	GFQK01289650.1	73.882	693	160	20	372	1055	1132	452	4.74E-67	257
249	maker-contig00716-fgenesh-gene-0.27-mRNA-1	GFQK01256347.1	77.898	371	74	8	22	388	213	579	1.97E-57	224
250	maker-contig00716-fgenesh-gene-0.27-mRNA-1	GFQK01083617.1	84.95	299	43	2	72	369	298	1	8.83E-81	302

S3 Table. (Cont'd)

Hit UI	Query sequence (*E. amarillans* transcriptome UI)	Subject sequence (creeping bent grass transcriptome UI)	Percentage of identical matches	Alignment length	Number of mismatch es	Number of gap openings	Start of alignment in query	End of alignment in query	Start of alignment in subject	End of alignment in subject	Expect value	Bit score
251	maker-contig00757-snap-gene-0.16-mRNA-1	GFQK01123154.1	80.561	499	86	9	979	1470	697	203	6.83E-102	374
252	maker-contig00757-snap-gene-0.16-mRNA-1	GFQK01274996.1	77.107	961	200	18	43	993	144	1094	2.29E-151	538
253	maker-contig00893-fgenesh-gene-0.12-mRNA-1	GFQK01269710.1	75.776	322	71	6	331	647	320	1	2.86E-36	156
254	maker-contig00936-fgenesh-gene-0.12-mRNA-1	GFQK01331191.1	77.344	256	56	2	184	438	255	1	8.86E-35	150
255	maker-contig01139-snap-gene-0.14-mRNA-1	GFQK01245506.1	80.747	348	64	3	926	1271	577	231	2.82E-70	268
256	snap_masked-contig00014-processed-gene-0.91-mRNA-1	GFQK01291212.1	85.634	355	46	4	1	354	143	493	6.55E-101	368
257	snap_masked-contig00025-processed-gene-0.44-mRNA-1	GFQK01283127.1	80	260	43	9	1347	1602	1407	1661	3.64E-44	183
258	snap_masked-contig00091-processed-gene-0.30-mRNA-1	GFQK01213989.1	80.913	241	28	7	1358	1598	226	4	8.64E-42	174
259	snap_masked-contig00091-processed-gene-0.30-mRNA-1	GFQK01116243.1	82.251	231	26	9	40	264	222	1	3.99E-45	185
260	snap_masked-contig00091-processed-gene-0.30-mRNA-1	GFQK01144782.1	78.977	352	41	11	260	608	1	322	2.37E-52	209
261	snap_masked-contig00091-processed-gene-0.30-mRNA-1	GFQK01077183.1	86.182	275	34	4	811	1083	273	1	6.35E-78	294
262	snap_masked-contig00091-processed-gene-0.30-mRNA-1	GFQK01165989.1	86.217	341	40	5	1086	1424	1	336	1.72E-98	363
263	snap_masked-contig00091-processed-gene-0.30-mRNA-1	GFQK01004526.1	84.856	383	55	1	659	1038	385	3	1.32E-104	383
264	snap_masked-contig00091-processed-gene-0.30-mRNA-1	GFQK01168980.1	82.915	995	143	11	778	1769	2	972	0	870
265	snap_masked-contig00091-processed-gene-0.30-mRNA-1	GFQK01011807.1	83.942	1314	188	10	457	1769	370	1661	0	1236
266	snap_masked-contig00102-processed-gene-0.14-mRNA-1	GFQK01309437.1	75.528	1042	229	22	5194	6219	1209	178	9.16E-136	488
267	snap_masked-contig00183-processed-gene-0.20-mRNA-1	GFQK01255836.1	87.339	466	57	2	1	465	677	213	2.98E-150	532
268	snap_masked-contig00305-processed-gene-0.22-mRNA-1	GFQK01143421.1	87.562	201	25	0	350	550	2	202	1.57E-59	233
269	snap_masked-contig00305-processed-gene-0.22-mRNA-1	GFQK01143421.1	83.853	1282	193	14	550	1824	277	1551	0	1208
270	snap_masked-contig00354-processed-gene-0.8-mRNA-1	GFQK01179659.1	80	190	36	2	79	267	430	242	4.13E-32	139
271	snap_masked-contig00484-processed-gene-0.20-mRNA-1	GFQK01133256.1	85.115	262	36	3	8	267	137	397	6.53E-70	265
272	snap_masked-contig00709-processed-gene-0.6-mRNA-1	GFQK01211553.1	81.707	246	43	2	685	929	247	3	7.60E-51	204
273	snap_masked-contig00807-processed-gene-0.11-mRNA-1	GFQK01050696.1	87.333	150	19	0	1995	2144	1	150	3.62E-41	172
