# Supplementary material for: Fungus-originated glucanase and monooxygenase genes in creeping bent grass (Agrostis stolonifera L.)
Source: PLoS One. 2021 Sep 10;16(9):e0257173. doi: 10.1371/journal.pone.0257173 (PMC8432771; doi:10.1371/journal.pone.0257173)
Supplement: S4 Table — (PDF) [file pone.0257173.s009.pdf]

S4 Table. BLAST DNA sequence similarity search using the *As*FMOL sequence as query.

Source organism	Kingdom	NCBI UI	Score		E-value	Identity
			Max	Total		
Epichloe festucae Fl1	Fungus	CP031391.1	2394	2394	0	95%
Metarhizium brunneum ARSEF 3297	Fungus	XM_014685347.1	891	891	0	78%
Metarhizium robertsii ARSEF 23	Fungus	XM_007824911.1	863	863	0	78%
Cordyceps militaris CM01	Fungus	XM_006673797.1	355	355	2E-93	73%
Metarhizium acridum CQMa 102	Fungus	XM_007810330.1	339	339	2E-88	77%
Cordyceps militaris	Fungus	CP023324.1	307	395	7E-79	74%
Pseudonocardia sp. AL041005-10	Bacteria	CP011862.1	156	156	3E-33	86%
Aspergillus candidus	Fungus	XM_024813143.1	148	148	4E-31	78%
Aspergillus campestris IBT 28561	Fungus	XM_024835573.1	137	190	1E-27	77%
Rhodococcus jostii RHA1	Bacteria	CP000431.1	115	115	5E-21	76%
Gordonia sp. YC-JH1	Bacteria	CP025435.1	104	104	1E-17	79%
Cordyceps militaris CM01	Fungus	XM_006671602.1	87.9	87.9	1E-12	75%
Thielavia terrestris NRRL 8126	Fungus	XM_003657523.1	78.7	78.7	6E-10	80%
Thielavia terrestris NRRL 8126	Fungus	CP003014.1	78.7	78.7	6E-10	80%
Phyllobacterium sp. 428-9	Bacteria	FJ263129.1	58.4	58.4	0.0008	91%
Uncultured bacterium	Bacteria	FJ263170.1	58.4	58.4	0.0008	87%
