# Supplementary material for: Fungus-originated glucanase and monooxygenase genes in creeping bent grass (Agrostis stolonifera L.)
Source: PLoS One. 2021 Sep 10;16(9):e0257173. doi: 10.1371/journal.pone.0257173 (PMC8432771; doi:10.1371/journal.pone.0257173)
Supplement: S5 Table — Read count quantification was preformed using the NCBI BLAST tool. The word size parameter was set at 64. The AsBGNL and AsFMOL sequences were used as query sequence. (PDF) [file pone.0257173.s010.pdf]

S5 Table. Short-read sequencing-based gene expression analysis.

Common name	Scientific name	NCBI SRA UI	# of Spots	# of reads	*As* FMOL		*As* BGNL	
					Hit number	FPKM	Hit number	FPKM
Creeping bentgrass	*Agrostis stolonifera*	SRX2962769	38,233,548	76,467,096	102	0.86	921	7.53
Hair grass	*Agrostis scabra*	SRX2582777-SRX2582785	40,162,225	80,324,450	25	0.20	717	5.58
-	*Deyeuxia angustifolia*	SRX692543	46,001,292	92,002,584	183	1.28	4643	31.54
