# Supplementary material for: Fungus-originated glucanase and monooxygenase genes in creeping bent grass (Agrostis stolonifera L.)
Source: PLoS One. 2021 Sep 10;16(9):e0257173. doi: 10.1371/journal.pone.0257173 (PMC8432771; doi:10.1371/journal.pone.0257173)
Supplement: S1 Raw images — (PDF) [file pone.0257173.s011.pdf]

Original image used for Fig 2a

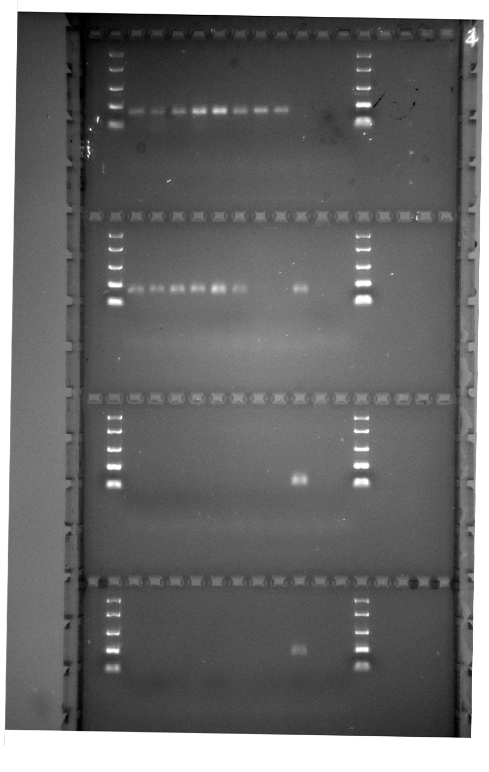

Row	Lane	PCR Primers	DNA template
1	1 (left)	(Empty)	
	2	(Size standard)	BIOLINE EasyLadder I
	3-5	AsBGNL/LpBGNL_F, AsBGNL_R	Creeping bent grass
	6-8	AsBGNL/LpBGNL_F, AsBGNL_R	Common bent
	9, 10	AsBGNL/LpBGNL_F, AsBGNL_R	Annual beard grass
	11	AsBGNL/LpBGNL_F, AsBGNL_R	*E. festucae*
	12	AsBGNL/LpBGNL_F, AsBGNL_R	Harding grass
	13	AsBGNL/LpBGNL_F, AsBGNL_R	NTC
	14	(Size standard)	BIOLINE EasyLadder I
	15-18 (right)	(Empty)	
2	1 (left)	(Empty)	
	2	(Size standard)	BIOLINE EasyLadder I
	3-5	AsFMOL_con_f1_AstII, AsFMOL_con_r1_AstII	Creeping bent grass
	6-8	AsFMOL_con_f1_AstII, AsFMOL_con_r1_AstII	Common bent
	9, 10	AsFMOL_con_f1_AstII, AsFMOL_con_r1_AstII	Annual beard grass
	11	AsFMOL_con_f1_AstII, AsFMOL_con_r1_AstII	*E. festucae*
	12	AsFMOL_con_f1_AstII, AsFMOL_con_r1_AstII	Harding grass
	13	AsFMOL_con_f1_AstII, AsFMOL_con_r1_AstII	NTC
	14	(Size standard)	BIOLINE EasyLadder I
	15-18 (right)	(Empty)	
3	1 (left)	(Empty)	
	2	(Size standard)	BIOLINE EasyLadder I
	3-5	C.purpurea_D0288F, C.purpurea_D0289R	Creeping bent grass
	6-8	C.purpurea_D0288F, C.purpurea_D0289R	Common bent
	9, 10	C.purpurea_D0288F, C.purpurea_D0289R	Annual beard grass
	11	C.purpurea_D0288F, C.purpurea_D0289R	*E. festucae*
	12	C.purpurea_D0288F, C.purpurea_D0289R	Harding grass
	13	C.purpurea_D0288F, C.purpurea_D0289R	NTC
	14	(Size standard)	BIOLINE EasyLadder I
	15-18 (right)	(Empty)	
4	1 (left)	(Empty)	
	2	(Size standard)	BIOLINE EasyLadder I
	3-5	Epichloe_mcf_F, Epichloe_mcf_R	Creeping bent grass
	6-8	Epichloe_mcf_F, Epichloe_mcf_R	Common bent
	9, 10	Epichloe_mcf_F, Epichloe_mcf_R	Annual beard grass
	11	Epichloe_mcf_F, Epichloe_mcf_R	*E. festucae*
	12	Epichloe_mcf_F, Epichloe_mcf_R	Harding grass
	13	Epichloe_mcf_F, Epichloe_mcf_R	NTC
	14	(Size standard)	BIOLINE EasyLadder I
	15-18 (right)	(Empty)	

Original image used for Fig 2b

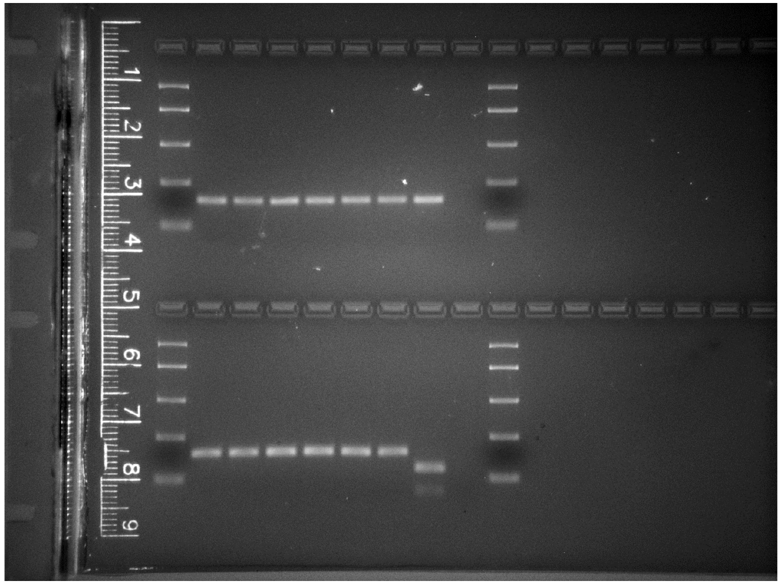

Row	Lane	PCR Primers	DNA template	*Aat* II
1	1 (left)	(Size standard)	BIOLINE EasyLadder I	
	2-4	AsFMOL_con_f1_AstII, AsFMOL_con_r1_AstII	Creeping bent grass	Untreated
	5-7	AsFMOL_con_f1_AstII, AsFMOL_con_r1_AstII	Common bent	Untreated
	8	AsFMOL_con_f1_AstII, AsFMOL_con_r1_AstII	*E. festucae*	Untreated
	9	AsFMOL_con_f1_AstII, AsFMOL_con_r1_AstII	NTC	Untreated
	10	(Size standard)	BIOLINE EasyLadder I	
	11-17 (right)	(Empty)		
2	1 (left)	(Size standard)	BIOLINE EasyLadder I	
	2-4	AsFMOL_con_f1_AstII, AsFMOL_con_r1_AstII	Creeping bent grass	Treated
	5-7	AsFMOL_con_f1_AstII, AsFMOL_con_r1_AstII	Common bent	Treated
	8	AsFMOL_con_f1_AstII, AsFMOL_con_r1_AstII	*E. festucae*	Treated
	9	AsFMOL_con_f1_AstII, AsFMOL_con_r1_AstII	NTC	Treated
	10	(Size standard)	BIOLINE EasyLadder I	
	11-17 (right)	(Empty)		

Original image used for S3 Fig

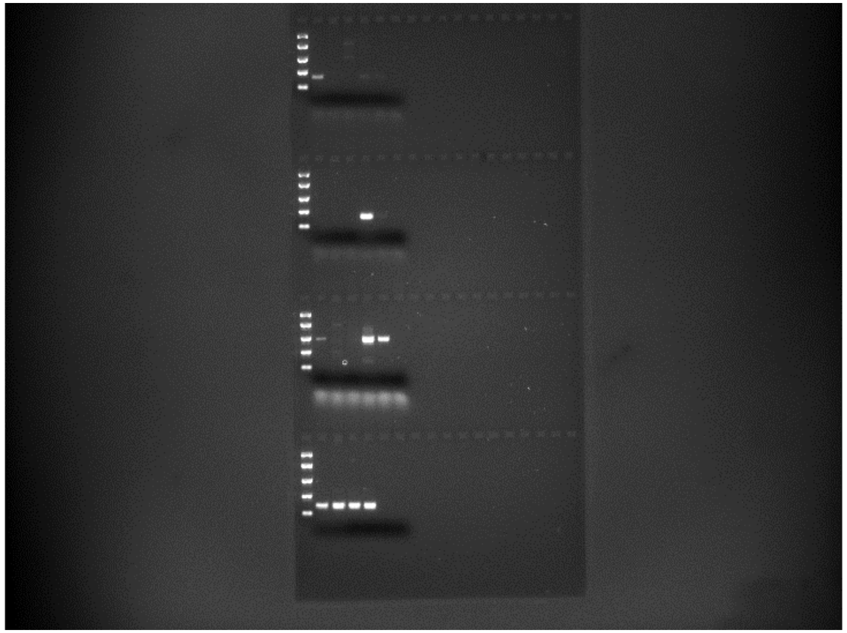

Row	Lane	PCR Primers	DNA template
1	1 (left)	(Size standard)	BIOLINE EasyLadder I
	2	AsBGNL/LpBGNL_F, AsBGNL_R	Creeping bent grass
	3	AsBGNL/LpBGNL_F, AsBGNL_R	Oat
	4	AsBGNL/LpBGNL_F, AsBGNL_R	Harding grass
	5	AsBGNL/LpBGNL_F, AsBGNL_R	Perennial ryegrass
	6	AsBGNL/LpBGNL_F, AsBGNL_R	*E. festucae*
	7	AsBGNL/LpBGNL_F, AsBGNL_R	NTC
	8-18 (right)	(Empty)	
2	1 (left)	(Size standard)	BIOLINE EasyLadder I
	2	AsBGNL/LpBGNL_F, LpBGNL_R	Creeping bent grass
	3	AsBGNL/LpBGNL_F, LpBGNL_R	Oat
	4	AsBGNL/LpBGNL_F, LpBGNL_R	Harding grass
	5	AsBGNL/LpBGNL_F, LpBGNL_R	Perennial ryegrass
	6	AsBGNL/LpBGNL_F, LpBGNL_R	*E. festucae*
	7	AsBGNL/LpBGNL_F, LpBGNL_R	NTC
	8-18 (right)	(Empty)	
3	1 (left)	(Size standard)	BIOLINE EasyLadder I
	2	LpBGNL_cons_F, LpBGNL_cons_R	Creeping bent grass
	3	LpBGNL_cons_F, LpBGNL_cons_R	Oat
	4	LpBGNL_cons_F, LpBGNL_cons_R	Harding grass
	5	LpBGNL_cons_F, LpBGNL_cons_R	Perennial ryegrass
	6	LpBGNL_cons_F, LpBGNL_cons_R	*E. festucae*
	7	LpBGNL_cons_F, LpBGNL_cons_R	NTC
	8-18 (right)	(Empty)	
4	1 (left)	(Size standard)	BIOLINE EasyLadder I
	2	FT-HD3_P_con_F, FT-HD3_P_con_R	Creeping bent grass
	3	FT-HD3_P_con_F, FT-HD3_P_con_R	Oat
	4	FT-HD3_P_con_F, FT-HD3_P_con_R	Harding grass
	5	FT-HD3_P_con_F, FT-HD3_P_con_R	Perennial ryegrass
	6	FT-HD3_P_con_F, FT-HD3_P_con_R	*E. festucae*
	7	FT-HD3_P_con_F, FT-HD3_P_con_R	NTC
	8-18 (right)	(Empty)	

Original images used for the upper panel of S5 Fig

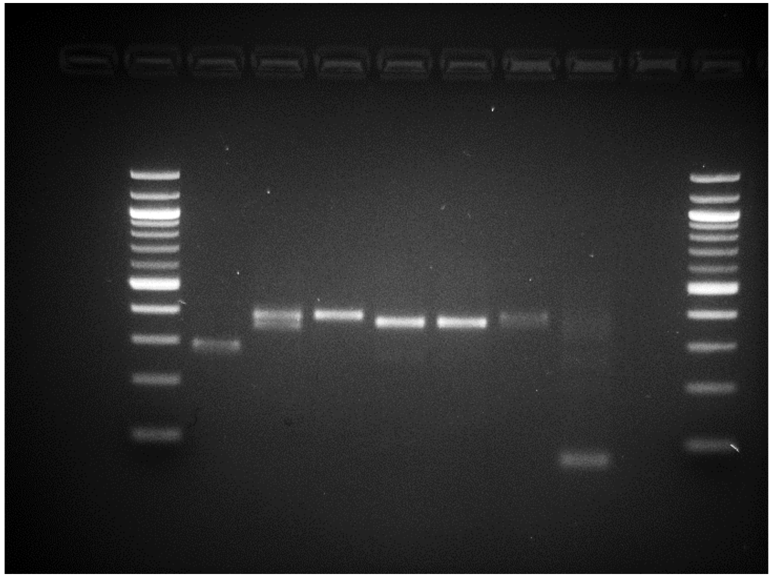

Lane	PCR Primers	DNA template
1 (left)	(Empty)	
2	(Size standard)	NEB 100 bp DNA Ladder
3-5	AsBGNL_exon_F, AsBGNL_exon_R	Creeping bent grass gDNA
6-8	AsBGNL_exon_F, AsBGNL_exon_R	Common bent gDNA
9	AsBGNL_exon_F, AsBGNL_exon_R	Creeping bent grass cDNA
10	AsBGNL_exon_F, AsBGNL_exon_R	NTC
11 (right)	(Size standard)	NEB 100 bp DNA Ladder

Original images used for the lower panel of S5 Fig

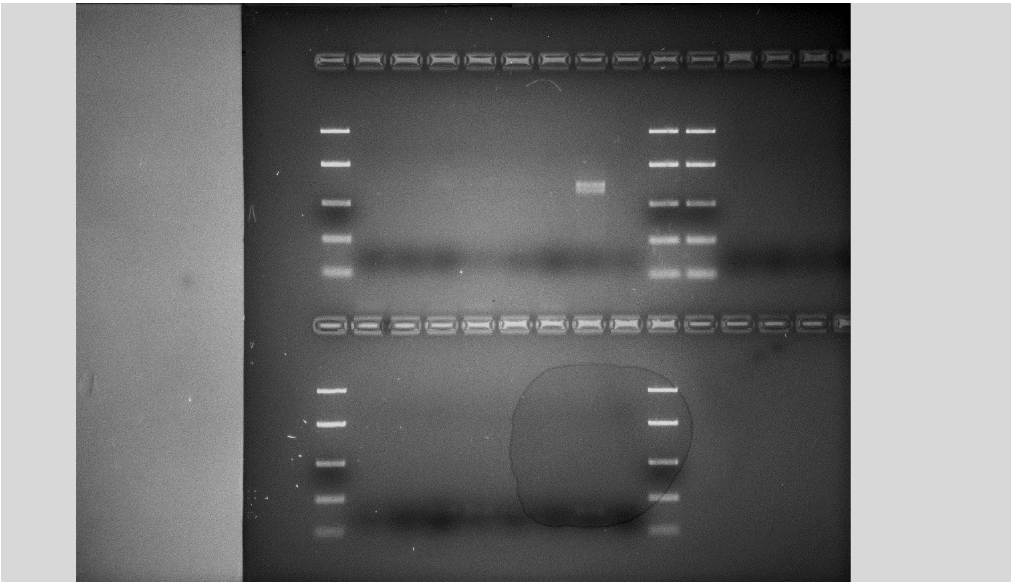

Row	Lane	PCR Primers	DNA template
1	1 (left)	(Size standard)	BIOLINE EasyLadder I
	2-4	AsBGNL_intron_F, AsBGNL_R	Creeping bent grass gDNA
	5-7	AsBGNL_intron_F, AsBGNL_R	Common bent gDNA
	8	AsBGNL_intron_F, AsBGNL_R	Creeping bent grass cDNA
	9	AsBGNL_intron_F, AsBGNL_R	NTC
	10	(Size standard)	BIOLINE EasyLadder I
	11-14 (right)	(Unrelated samples)	
2	1-14	(Unrelated samples)	
