# Supplementary material for: Fungus-originated glucanase and monooxygenase genes in creeping bent grass (Agrostis stolonifera L.)
Source: PLoS One. 2021 Sep 10;16(9):e0257173. doi: 10.1371/journal.pone.0257173 (PMC8432771; doi:10.1371/journal.pone.0257173)
Supplement: Attachment — Submitted filename: Response to Reviewers.docx [file pone.0257173.s012.docx]

Dear Editor and Reviewers,

We would like to appreciate the Reviewers for their generous comments on the manuscript. The followings are point-to-point responses to the Reviewers.

**Comment 1 from Reviewer #1:** *As there is no functional analysis, the last paragraph "adaptation mechanisms of flowering plants (Angiosperms)" is an over interpretation from the present results.*

**Response for comment 1:** The sentence was corrected following the comment (Lines 329-330 of the marked-up copy). How horizontally transferred genes contributed to recipient’s adaptation is one of current argument in this field. A citation was added (Dunning et al. 2019 doi:10.1073/pnas.1810031116).

**Comment 2 from Reviewer #1:** *Fig.1a As Perennial ryegrass is not the main focus of this study, it is better to place it in the third lane.Furthermore, is there a clear reason why Fig.1a should include the amino acid sequence of perennial ryegrass and Fig.1b should contain the amino acid sequence of E. festucae? Is there a problem with comparing only E. amarillans and Creeping bent grass?*

**Response for comment 2:** The alignment was from the CLUSTALW program, and no manual adjustment was performed. The reason why perennial ryegrass sequence is included in the AsBGNL figure is that the perennial ryegrass sequence showed a higher sequence identity with AsBGNL than the E. amarillans sequence did. Regarding the AsFMOL figure, E. festucae is a representative species of Epichloe and the genome of E. festucae has been better assembled than other Epichloe species in the database (Schardl et al. 2013 doi:10.1371/journal.pgen.1003323). As Epichloe genomes in the database have not been completely assembled, we believe that inclusion of multiple data points is essential, to eliminate a possibility of sequencing contamination or assembly artifacts.

**Comment 3 from Reviewer #1:** *L241-L244 I could not understand why it is unsuitable for calculating relative gene expression in AsFMOL. Why not just increase the number of cycles?*

**Response for comment 3:** A common question for an HGT candidate is whether the gene is expressed in the recipient species. The main purpose of the experiment is to determine this. In a qPCR assay, higher Cq values are generally less reliable, due to generation of some amounts of PCR artifacts (primer dimers and non-specific product) and a higher sample variance (Ruiz-Villalba et al. 2021 doi:10.3390/life11060496; Taylor et al. 2019 doi:10.1016/j.tibtech.2018.12.002). Therefore, the cutoff value should be set to somewhere between 30-35 cycles. In our case, the Cq values from more than half samples were over 35 cycles, to conclude that the qPCR result was not suitable for the relative expression analysis. We also tried other PCR primer combinations, to find that none of them worked better than the AsFMOL_ps_f1 and r1 combination. The references for the qPCR QC process have been added in the text (Line 243 of the marked-up copy).

**Comment 4 from Reviewer #1:** *L269 fine--leaves -> fine-leaves, L307 in cool cool-season grasses -> in cool-season grasses*

**Response for comment 4:** The text was corrected following the comments.

**Comment from Reviewer #2:** *Some references are not according to the journal format, it has to be modified accordingly.*

**Response for comment:** The reference list was generated the Zotero program (<https://www.zotero.org>). Then, the list was manually corrected, which can be found in Lines 346-436 of the marked-up copy.

**Comment from Reviewer #3:** *There are previously published reports regarding the lateral gene transfer in grasses. Authors should discuss in more details regarding the major outcome of this research and highlight its importance.*

**Response for comment:** Discussion about the recent progress in grass species, especially in Panicoideae subfamily was inserted, which can be found Lines 296-311 of the marked-up copy. For this section, two references (Dunning et al. 2019 doi:10.1073/pnas.1810031116; Hibdige et al. 2021 doi:10.1111/nph.17328) were added

I believe that all points raised by the Reviewers have been addressed.

Hiroshi Shinozuka

On behalf of all authors.
